# The fork protection complex promotes parental histone recycling and epigenetic memory

**DOI:** 10.1016/j.cell.2024.07.017

**Published:** 2024-09-05

**Authors:** Sebastian Jespersen Charlton, Valentin Flury, Yutaka Kanoh, Aitana Victoria Genzor, Leonie Kollenstart, Wantong Ao, Peter Brøgger, Melanie Bianca Weisser, Marek Adamus, Nicolas Alcaraz, Charlotte M. Delvaux de Fenffe, Francesca Mattiroli, Guillermo Montoya, Hisao Masai, Anja Groth, Geneviève Thon

**Affiliations:** 1 Department of Biology, University of Copenhagen, Copenhagen 2200, Denmark; 2 Novo Nordisk Foundation Center for Protein Research, University of Copenhagen, Copenhagen 2200, Denmark; 3 Tokyo Metropolitan Institute of Medical Science, Tokyo 156-8506, Japan; 4 Hubrecht Institute-KNAW & University Medical Center Utrecht, Utrecht, The Netherlands; 5 Biotech Research & Innovation Centre, University of Copenhagen, Copenhagen 2200, Denmark; 6 Department of Cellular and Molecular Medicine, University of Copenhagen, Copenhagen 2200, Denmark

**Keywords:** heterochromatin, DNA replication, histone recycling, histone chaperone, H3K9 methylation, chromatin replication, epigenome maintenance, epigenetic inheritance, fission yeast, mouse embryonic stem cells, Claspin

## Abstract

The inheritance of parental histones across the replication fork is thought to mediate epigenetic memory. Here, we reveal that fission yeast Mrc1 (CLASPIN in humans) binds H3-H4 tetramers and operates as a central coordinator of symmetric parental histone inheritance. Mrc1 mutants in a key connector domain disrupted segregation of parental histones to the lagging strand comparable to Mcm2 histone-binding mutants. Both mutants showed clonal and asymmetric loss of H3K9me-mediated gene silencing. AlphaFold predicted co-chaperoning of H3-H4 tetramers by Mrc1 and Mcm2, with the Mrc1 connector domain bridging histone and Mcm2 binding. Biochemical and functional analysis validated this model and revealed a duality in Mrc1 function: disabling histone binding in the connector domain disrupted lagging-strand recycling while another histone-binding mutation impaired leading strand recycling. We propose that Mrc1 toggles histones between the lagging and leading strand recycling pathways, in part by intra-replisome co-chaperoning, to ensure epigenetic transmission to both daughter cells.

## Highlights

• Mrc1 binds histone H3-H4 tetramers
• Mrc1 is required for efficient recycling to both daughter strands
• Mrc1 acts with Mcm2 in recycling to the lagging strand via co-chaperoning of H3-H4
• Mrc1 recycling mutants show clonal and asymmetric loss of H3K9me-mediated silencing

## Graphical abstract

**Figure:**
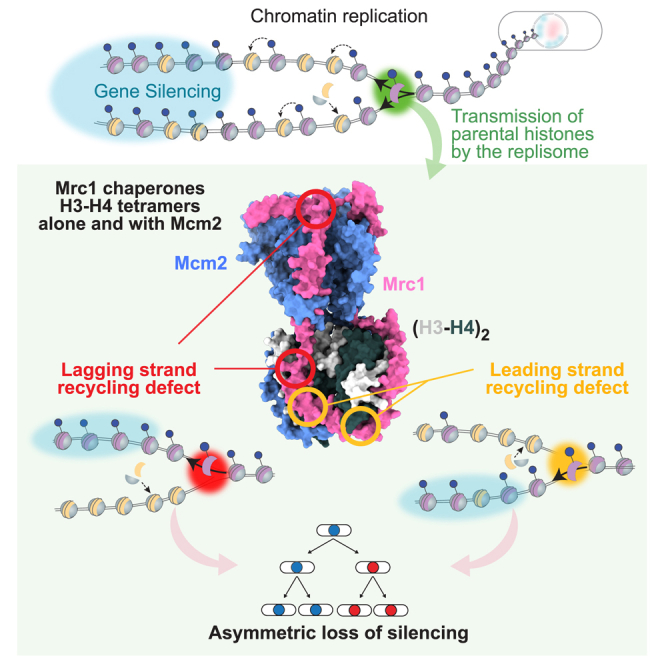

## Teaser

The replisome component Mrc1 chaperones H3-H4 tetramers, alone or with Mcm2, and facilitates parental histone transmission to leading and lagging DNA strands and epigenetic silencing.

## Introduction

In eukaryotes, DNA replication is accompanied by the coordinated duplication of chromatin structures to ensure faithful transmission of specialized chromatin states to daughter cells.^1^ This is critical for maintenance of cell identity in development and to counteract changes in cell function associated with aging and diseases like cancer. Recent advances have identified histone-binding capacities in the replisome responsible for transmission of histone-based information. However, it remains a fundamental question how the replisome coordinates fork progression with faithful inheritance of epigenetic states.

**Figure 1:**
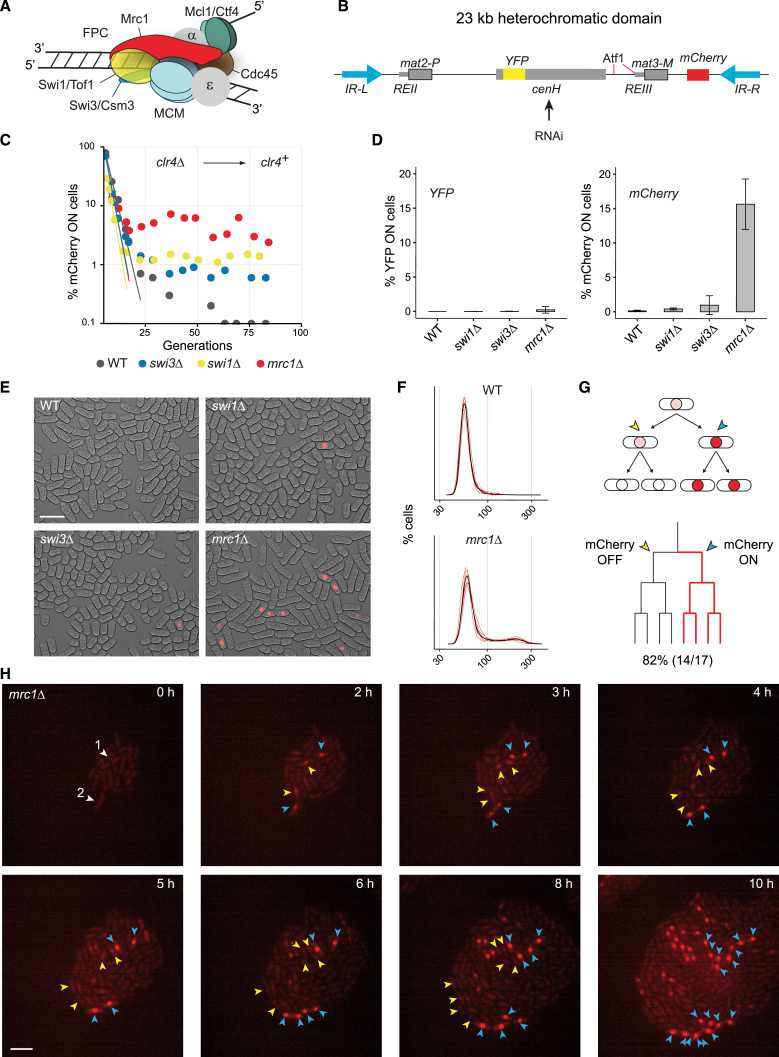
The FPC is essential for the maintenance of heterochromatic gene silencing (A) Illustration of the FPC (Mrc1, Swi1/Tof1, Swi3/Csm3) at the replisome, showing the CMG helicase (MCM2-6, Cdc45), Mcl1/Ctf4, DNA primase (Polα), and polymerase epsilon (Polε). (B) Heterochromatic mating-type region between the *IR-L* and *IR-R* boundaries depicting the silent mating-type cassettes *mat2-P* and *mat3-M*, *Kint2::YFP* and *(EcoRV)::mCherry* fluorescent reporter genes, RNAi-dependent nucleation center *cenH*, Atf1 transcription factor binding sites, and *REII* and *REIII* silencing elements. (C) Establishment of *mCherry* silencing following *clr4*^+^ reintroduction in FPC mutants. (D) Cells with derepressed reporters in clonal cultures of FPC mutants (*n* = 6). Data are represented as mean ± SD. (E) Micrographs of FPC mutants expressing *mCherry*. Scale bar: 10 μm. (F) Histograms of *mCherry* fluorescence (mean, black; replicates *n* = 6, red). (G) Interpretation of loss-of-silencing events in (H). The *mCherry* locus asymmetrically loses heterochromatic silencing in S phase, and the protein production starts in G2 (top cell). The expressed (*mCherry* ON) and repressed (*mCherry* OFF) chromatids segregate to sister cells. In subsequent cell divisions, mCherry protein is produced in the ON lineage (blue arrows) but is diluted in the OFF lineage (yellow arrows). (H) Loss of silencing in the *mrc1*Δ mutant followed by time-lapse microscopy. White arrowheads point to cells experiencing a loss-of-silencing event. Blue arrowheads point to ON cell lineages and yellow arrows point to OFF lineages. Yellow arrows are omitted at 10 h for clarity. Scale bar: 10 μm.

Structural studies of the replisome have revealed the position of the fork protection complex (FPC) constituted by Mrc1 (CLASPIN in humans), Tof1 (Timeless in humans; Swi1 in fission yeast), and Csm3 (Tipin in humans; Swi3 in fission yeast). Tof1/Timeless and Csm3/Tipin grip DNA at the front of the replisome, while the unstructured Mrc1/CLASPIN protein stretches across the replisome, making numerous contacts with Mcm2, Mcm6, and Ctf4^2^^,^^3^^,^^4^ (Figure 1A). This provides a framework to understand FPC function, such as regulation of fork progression,^5^^,^^6^^,^^7^^,^^8^^,^^9^^,^^10^^,^^11^ recognition of programmed replication blocks,^9^^,^^12^ and stabilization of stalled replication forks.^7^^,^^13^^,^^14^ Under replication stress, Mrc1 phosphorylation by the Tel1 and Rad3 kinases stabilizes replication forks and prevents the firing of late replication origins.^15^^,^^16^^,^^17^^,^^18^^,^^19^^,^^20^^,^^21^ Fission yeast Mrc1 also regulates the firing of early replication origins in unperturbed S phase through interaction with and phosphorylation by Hsk1 kinase (Cdc7 in other organisms).^22^^,^^23^

Intriguingly, fission yeast FPC mutants have appeared in large-scale genetic screens for heterochromatin defects.^24^^,^^25^^,^^26^^,^^27^^,^^28^ Fission yeast heterochromatin fulfills structural and regulatory roles at centromeres, telomeres, mating-type region, and other chromosomal locations. As for higher eukaryotes, heterochromatic nucleosomes are methylated at histone H3K9 (H3K9me) by an SUVAR39 family enzyme, Clr4.^29^ Shortly after DNA replication, a brief wave of transcription occurs at repeats present at heterochromatic locations, producing non-coding transcripts that fuel RNA interference (RNAi).^30^^,^^31^ Together with region-specific DNA elements and cognate binding proteins,^32^^,^^33^^,^^34^^,^^35^ RNAi induces the deposition of H3K9me.^36^ This is facilitated by positive feedback through read/write interactions.^37^^,^^38^ Feedback occurs through chromodomain proteins such as Swi6 (HP1) and Clr4 itself^39^^,^^40^^,^^41^^,^^42^ whose chromodomains bind H3K9me-modified nucleosomes to recruit enzymatic activities fostering more of the same modifications.^43^^,^^44^ The point of action of the FPC in this multi-step process is unknown.

Replisome components can mediate recruitment of nucleosome assembly factors, histone modifying enzymes, and the recycling of modified parental histones. Fission yeast DNA polymerase ε interacts with the Clr4 complex^45^ and histone deacetylases (HDACs),^46^ and Mrc1 interacts with the HDAC Clr3.^47^ Yet, it is unclear how recruitment to the generic replisome would maintain local chromatin structures. To this end, the replisome promotes an even distribution of parental and newly synthesized histones on daughter DNA strands so that parental histones can template the modification of new histones through read/write mechanisms. For example, Mcm2 chaperones parental histone H3-H4 tetramers and facilitates their transmission to the lagging strand.^48^^,^^49^^,^^50^^,^^51^ Mcm2 cooperates with Polα and Ctf4 for histone transfer to the lagging strand, the suggested Mcm2-Ctf4-Polα axis.^49^^,^^50^^,^^52^^,^^53^ The Mcm2 histone-binding domain (HBD) is necessary for silencing in budding yeast,^50^^,^^52^ fission yeast,^54^ and mammalian cells.^55^^,^^56^ Conversely, the Dpb3/4 subcomplex of DNA polymerase ε (POLE3/4 in mammals) chaperones parental histones toward the leading strand.^53^^,^^57^ Histones H2A-H2B are also transmitted symmetrically across the replisome in a manner that involves Polα.^58^^,^^59^ Dynamic chaperones such as FACT and Asf1 may facilitate the movement of histones across the replisome by concomitant histone binding, so called co-chaperoning, with replisome factors.^48^^,^^51^^,^^52^^,^^60^ Tof1 facilitates FACT recruitment^61^^,^^62^ and its co-chaperoning of an H3-H4 tetramer with MCM2,^63^^,^^64^ enabling progression of the replisome on a nucleosome template.^65^ However, our understanding of histone transmission across the replisome, including its path and transfer mechanisms, remains rudimentary.

To uncover FPC function in epigenetic inheritance, we combined genetics and tailored genomics in fission yeast. We identified a checkpoint- and origin-firing-independent function of Mrc1 in heterochromatin maintenance that requires histone and Mcm2 binding by a connector domain. Mrc1 mutants lacking this domain and Mcm2 HBD mutants alike failed to segregate parental histones to the lagging strand and showed asymmetric inheritance of silencing in daughter cells. Guided by AlphaFold predictions, we found that Mrc1 binds H3-H4 tetramers and that Mrc1 and Mcm2 act together, co-binding H3-H4 tetramers to facilitate their transmission to the lagging strand. This direct cooperation between two replisome components in histone handling provides a mechanism for moving H3-H4 across the replisome independent of soluble chaperones. Moreover, analysis of different Mrc1 histone-binding mutants revealed functions also in leading strand recycling, placing Mrc1 as a master regulator with the possibility to toggle histones between leading and lagging strands.

## Results

### The FPC is essential for faithful heterochromatin maintenance

To characterize the dynamics of heterochromatin formation and stability in FPC mutants, we first used the mating-type region, a classical model for epigenetic inheritance. Heterochromatin is established at the *cenH* element through RNAi and then spreads stochastically to the entire region in a process facilitated punctually by Atf1-binding sites and by the domain boundaries *IR-L* and *IR-R*^66^^,^^67^ (Figure 1). Once established, it can be maintained for hundreds of cell divisions in the absence of RNAi^36^^,^^68^ or *cenH*.^69^^,^^70^ This can be monitored at the single-cell level with fluorescent reporters.^34^^,^^66^^,^^67^^,^^71^ We monitored RNAi activity with *YFP* introduced at *cenH* (*kint2::YFP*) and heterochromatin propagation and maintenance with *mCherry* introduced at a peripheral location (*(EcoRV)::mCherry*; Figure 1B).

We first compared the rates of *de novo* heterochromatin formation in *swi1*Δ, *swi3*Δ, and *mrc1*Δ mutants with wild type (WT) by reintroducing a functional *clr4*^+^ gene into *clr4*Δ cells that completely lack H3K9me heterochromatin. The proportion of cells expressing *mCherry* decayed exponentially over time with nearly identical rates in all strains (Figure 1C). The FPC is therefore not required for heterochromatin establishment at the mating-type region. However, a proportion of *swi1*Δ, *swi3*Δ, and *mrc1*Δ mutant cells failed to repress *mCherry* at late timepoints after >25 generations*,* with *mrc1*Δ mutants showing the strongest silencing defect (Figure 1C). Clonal cultures showed similar bimodal populations with small proportions of cells expressing *mCherry* (Figures 1D–1F) while *Kint2::YFP* was silenced in all cells (Figure 1D).

Time-lapse microscopy showed both loss and gain of *mCherr*y silencing during micro-colony formation. A remarkable pattern of asymmetric derepression was observed where two sister cells initially showed an mCherry signal; the derepressed state was propagated in one lineage while the signal rapidly decayed and remained off in the other lineage (Figures 1G and 1H). The pattern suggested that heterochromatin loss often occurs asymmetrically on just one of the two sister chromatids. Expression from that chromatid would lead to a brief accumulation of mCherry protein in G2 prior to cell division, but only the descendants of the cell inheriting the derepressed chromatid would continue expression.

**Figure S1:**
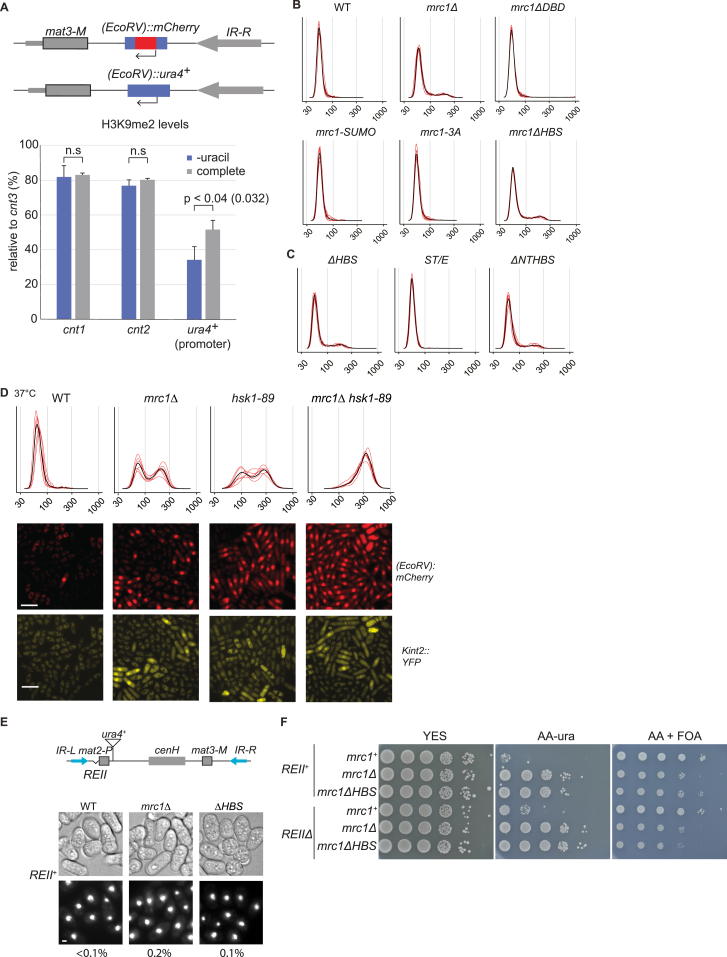
Mrc1 maintains heterochromatin independently of its functions in genome stability and origin regulation, related to Figure 2 (A) H3K9me2 levels in *mrc1ΔHBS* mutants with an *(EcoRV)::ura4*^*+*^ reporter. *(EcoRV)::ura4*^*+*^ and *(EcoRV)::mCherry* differ only at their ORF. *(EcoRV)::ura4*^*+*^ was used to select for cells in the expressed state by propagating them in EMM2 medium lacking uracil. Statistics using unpaired Student’s t test. Average of three independent replicates. H3K9me2 was analyzed by ChIP-seq. The bar-diagram shows H3K9me2 levels quantified by ChIP-seq normalized to input and shown relative to signal at *cnt3*. (B and C) Histograms of mCherry cell fluorescence intensities used to generate Figure 2B B and Figure 2C C. (D) Histograms of mCherry cell fluorescence intensities used to generate Figure 2G and representative micrographs. Note that some *hsk1-89* cells displayed autofluorescence rather than the expected nuclear signal for YFP expression. Scale bar: 10 μm. (E) Mrc1 functions redundantly with the *REII* element to repress *mat2-P* mating-type information, dependent on the HBS domain. The proportion of cells undergoing haploid meiosis is shown as in Figure 2H but for the *REII*^*+*^ control. Scale bar: 1 μm. (F) Mrc1 and HBS domain are necessary for the repression of the *(XbaI)::ura4*^*+*^ reporter gene near *mat2-P*, redundantly with the *REII* element. Ten-fold serial dilutions of cell suspensions were spotted onto the indicated media. Growth on AA-ura reflects *ura4*^*+*^ expression while growth on the toxigenic substrate FOA reflects *ura4*^*+*^ repression.

To investigate the chromatin state of derepressed cells, we turned to the *(EcoRV)::ura4*^*+*^ reporter. By selecting for cells that express *(EcoRV)::ura4*^*+*^ in medium lacking uracil, we observed reduced H3K9me2 at the *ura4*^*+*^ promoter (Figure S1A).

Altogether, we concluded that the FPC is required for heterochromatin maintenance rather than establishment, with a stronger contribution by Mrc1 than by Swi1 or Swi3.

### Mrc1 maintains heterochromatin independently of its functions in genome stability and origin regulation

**Figure 2:**
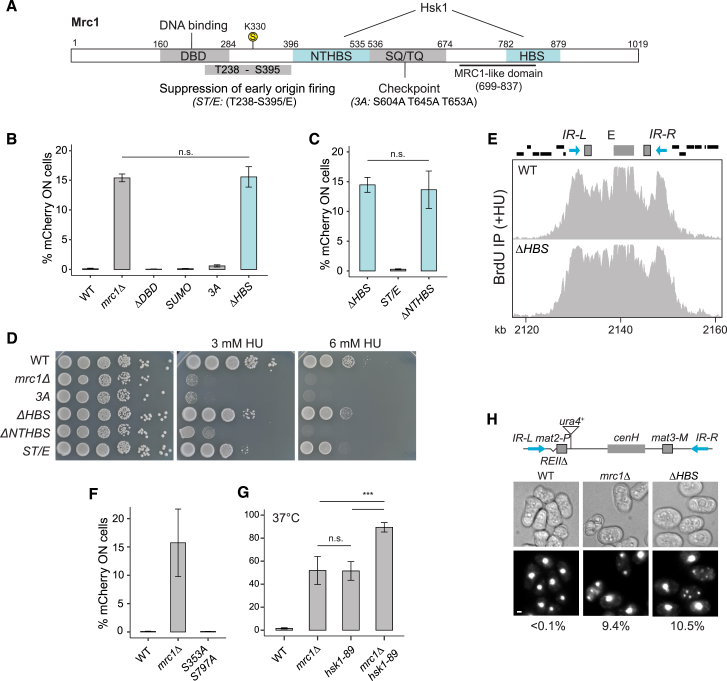
Mrc1 maintains heterochromatin independently of its functions in genome stability and origin regulation (A) Mrc1 protein with annotated domains and residues. MRC1-like domain refers to the most conserved portion of Mrc1, Pfam domain PF09444. (B and C) Cells expressing *mCherry* (*n* = 6). (B) [ANOVA, F = 654, *p* = 2.22 × 10^−16^]; (C) [ANOVA, F = 99, *p* = 2.13 × 10^−9^]. Data are represented as mean ± SD. (D) Checkpoint proficiency of *mrc1* mutants. 10-fold serial dilutions of cell suspensions were spotted onto HU-containing YES plates and incubated at 33°C for 4 days. (E) BrdU incorporation profiles in the mating-type region in cells released into S phase in the presence of HU. (F and G) Cells expressing *mCherry* (F, *n* = 3; G, *n* = 6). (G) Cells were propagated at the permissive temperature for *hsk1-89*, 37°C, which inherently weakens heterochromatin seen by slight loss of *mCherry* silencing even in WT cells. [ANOVA, F = 135, *p* = 1.88 × 10^−13^]. Data are represented as mean ± SD. (H) Haploid meioses in *mrc1*Δ and *mrc1*Δ*HBS* mutants lacking the *REII* silencing element visualized by bright field imaging and Hoechst staining of cells propagated on MSA sporulation medium. Scale bar: 1 μm. See also Figure S1.

We took advantage of separation-of-function mutants to identify the Mrc1 function relevant to heterochromatin maintenance (Figure 2A). Replacing three S/T residues in Mrc1 abolishes Mrc1 regulation by the replication checkpoint and causes hydroxyurea (HU) sensitivity (S604, T645, T653; *mrc1-3A* mutant).^17^ *mCherry* remained strongly repressed in the *mrc1-3A* mutant (Figures 2B and S1B), showing that Mrc1 stabilizes heterochromatin independently of its checkpoint functions. Mutants lacking the Mrc1 DNA-binding domain (amino acids [aa] 160–284)^72^ or a sumoylation site of Mrc1 at K330^72^ were also proficient for silencing (Figure 2B). Two regions, the Hsk1-bypass segment (HBS, aa 782–879) and N-terminal target of HBS (NTHBS, aa 396–535)^22^ proved crucial as their loss reproduced the silencing defect of *mrc1* deletion (Figures 2B, 2C, S1B, and S1C). The checkpoint is functional in the HBS mutant but not in the NTHBS mutant (Figure 2D).^22^ Thus, the *mrc1*Δ*HBS* mutation separates the silencing function from checkpoint functions and identifies the HBS domain as key to Mrc1 function in heterochromatin.

Since the HBS domain regulates replication origins in unperturbed S phase,^22^ we asked whether Mrc1 function in heterochromatin involved checkpoint-independent suppression of early-origin firing. Like the HBS mutant, an *mrc1* mutant with all serine and threonine residues substituted to aspartate in the T238–S395 interval (*mrc1-ST/E*) is defective in early-origin suppression,^22^ but that mutant did not show a heterochromatin maintenance defect (Figure 2C). Moreover, the characteristic early replication profile of the mating-type region^73^ was unaltered in the *mrc1ΔHBS* mutant (Figure 2E), further arguing that the heterochromatic defect is not caused by altered origin usage.

The HBS and NTHBS domains physically and functionally interact with the Hsk1 kinase,^22^ and Hsk1 phosphorylates Mrc1 at S353 and S797.^74^ However, an S353A S797A mutant failed to produce a silencing defect (Figure 2F). Second, the *mrc1*Δ and the temperature-sensitive *hsk1-89* allele^75^ showed additive effects on loss of silencing (Figures 2G and S1D). We concluded that Mrc1 and Hsk1 have independent modes of action in heterochromatic gene silencing.

The *mrc1*Δ*HBS* silencing defect was confirmed using a sporulation phenotype (Figure 2H and S1E). Heterochromatin defects that permit expression of mating-type information from *mat2-P* or *mat3-M* result in meiotic induction in haploid cells, termed “haploid meiosis.” This is a lethal event naturally prevented by redundant mechanisms. Using a tester strain lacking the *REII* element which silences *mat2-P* redundantly with H3K9me,^76^^,^^77^ we detected haploid meioses in *mrc1*Δ and *mrc1*Δ*HBS* mutants (Figure 2H and S1E). The *(XbaI)::ura4*^*+*^ reporter gene located next to *mat2-P* was also derepressed in the mutants, both in *REII*^*+*^ and *REIIΔ* cells (Figure S1F), altogether showing widespread loss of silencing in the *mrc1*Δ*HBS* mutant.

### Concerted function of Mrc1 and Mcm2 in heterochromatin maintenance

**Figure 3:**
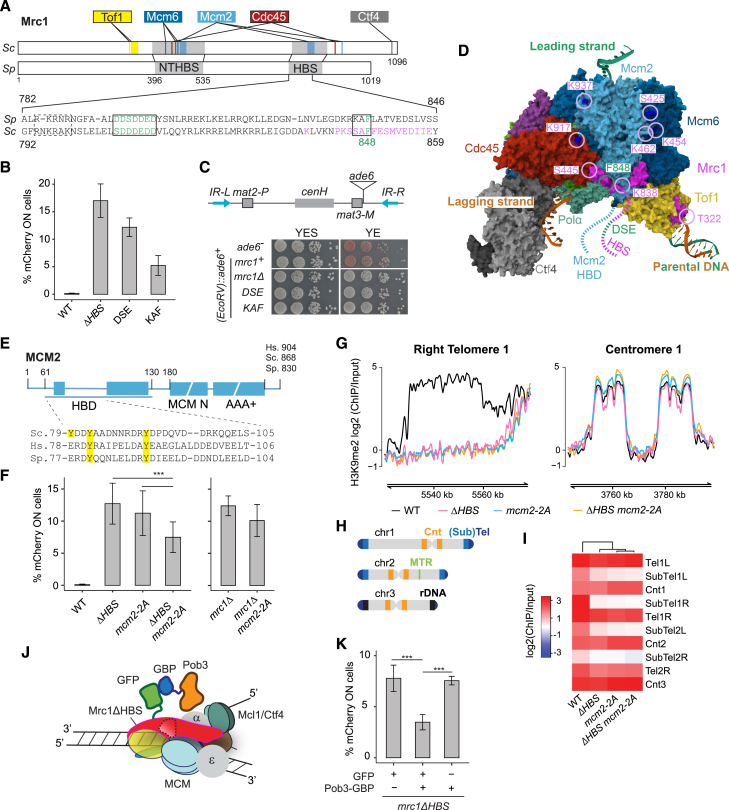
Coordinated function of Mrc1 and Mcm2-HBD in heterochromatin maintenance (A) Aligned *S. cerevisiae* and *S. pombe* Mrc1 proteins showing contacts and cross-links to replisome components in *S. cerevisiae*^2^^,^^4^ and highly conserved residues in Mrc1 HBS (bottom). Boxed residues were mutated in this study. Magenta residues are identified in the cryo-EM structure.^4^ (B) Cells expressing *mCherry* (*n* = 6). Data are represented as mean ± SD. (C) Expression of *ade6*^*+*^ reporter in *mrc1* mutants. Red color on YE medium indicates repression. (D) Mrc1 contacts with replisome components shown in magenta on PDB 8B9A^4^ with the expected positions of the Mcm2 HBD and Mrc1 HBS and DSE indicated. Cross-linked residues (from Baretić et al. [2020]^2^) are indicated by magenta circles and labeled by their position in *S. cerevisiae* Mrc1. The conserved F848 in KAF is in green. (E) Conserved tyrosine residues in the Mcm2 HBD mutated to alanine in *mcm2-2A*. (F) Cells expressing *mCherry* (*n* = 18 [left] [ANOVA, F = 73.9, *p* = 2.2 × 10^−^^16^]; *n* = 6 [right]). Data are represented as mean ± SD. (G) H3K9me2 in subtelomeric region Tel1R in *mrc1*Δ*HBS and mcm2-2A* mutants. Centromere 1 is shown for comparison. (H) Major heterochromatic regions of *S. pombe*. (I) Heatmap depicting H3K9me2 at heterochromatic regions. (J and K) Tethering Pob3 to Mrc1ΔHBS through a GFP-GBP interaction (J) restores silencing of *mCherry* reporter (K). (*n* = 6) [ANOVA, F = 45.9, *p* = 7.1 × 10^−^^7^]. Data are represented as mean ± SD. See also Figures S2 and S3.

**Figure S2:**
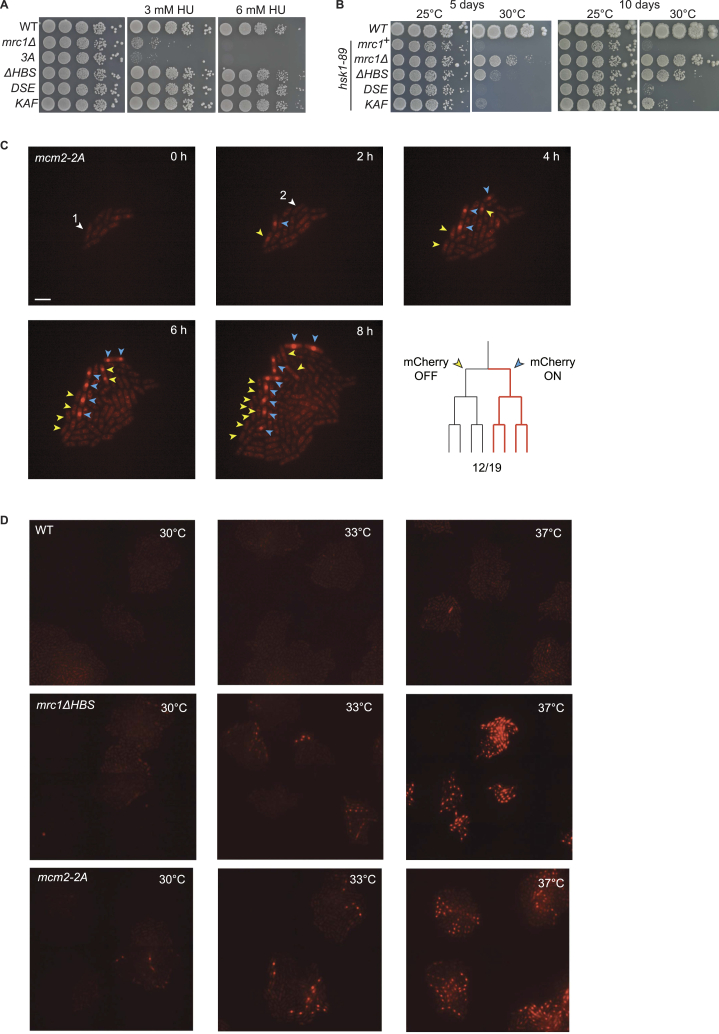
Proficient checkpoint in mrc1-DSE and mrc1-KAF mutants and similarities of mrc1ΔHBS and mcm2-2A mutants, related to Figure 3 (A) Ten-fold serial dilutions of cell suspensions were spotted on HU-containing medium to estimate checkpoint proficiency. (B) Ten-fold serial dilutions of cell suspensions were spotted on rich YES medium and incubated at the indicated temperatures to check for bypass of the *hsk1-89* mutation at the restrictive temperature of 30°C. (C) Asymmetric loss of heterochromatic gene silencing of *(EcoRV)::mCherry* reporter in *mcm2-2A* mutant visualized by time-lapse microscopy. Scale bar: 10 μm. (D) Similar temperature-dependency of heterochromatic silencing in *mrc1*Δ*HBS* and *mcm2-2A* mutants. In both (C) and (D), microcolonies were grown from single cells under a fluorescence microscope.

Within the Mrc1 HBS domain, aa 795–801 (DDSDDED) and 833–835 (KAF) show high sequence conservation among yeasts and other eukaryotes (Figure 3A).^22^ Alanine substitutions at these residues (DDSDDED in *mrc1-DSE* and KAF in *mrc1-KAF*) impaired silencing (Figure 3B) without affecting the replication checkpoint (Figure S2A), and *mrc1-KAF*, but not *mrc1-DSE*, partially bypassed the requirement for Hsk1 at 30°C (Figure S2B). Loss of silencing was also evident using a standard, but not quantitative, *ade6*^*+*^ reporter where loss of repression leads to white colonies (Figure 3C).

When mapping contact points detected by cross-linking mass spectrometry (XL-MS) or cryoelectron microscopy (cryo-EM) between replisome factors and *S. cerevisiae* Mrc1^2^^,^^4^ onto *S. pombe* Mrc1, most contacts fall within the NTHBS or HBS domains and many involve Mcm2 (Figure 3A). The contacts between Mcm2 and Mrc1^2^^,^^4^ suggested a physical proximity of the Mrc1 DDSDDED and KAF motifs and the amino-terminal domain of Mcm2 at the front of the replicative helicase (Figure 3D). Given the role of the Mcm2 amino-terminal HBD in parental histone recycling^49^^,^^50^ and the asymmetric loss of silencing in *mrc1* mutants (Figures 1G and 1H), Mrc1 might participate in histone recycling together with Mcm2. To address this, we created a fission yeast Mcm2 HBD mutant by alanine substitution of two conserved tyrosines, Y80 and Y89, essential for histone binding in other species^51^^,^^52^^,^^60^ (*mcm2-2A*, Figure 3E). The *mcm2-2A* mutation led to derepression of the *mCherry* reporter (Figures 3F, S2C, and S2D). Like the *mrc1* mutants, derepression occurred in a fraction of the population, produced the same asymmetric pattern of silencing loss in time-lapse microscopy (Figure S2C), and showed similar temperature dependency (Figure S2D). Moreover, double *mrc1*Δ *mcm2-2A* and *mrc1*Δ*HBS mcm2-2A* mutants showed a slight suppression of the silencing defect compared to single mutants (Figure 3F). Together, this suggested that Mrc1 and Mcm2 contribute to heterochromatin maintenance through a shared mechanism (see Discussion).

**Figure S3:**
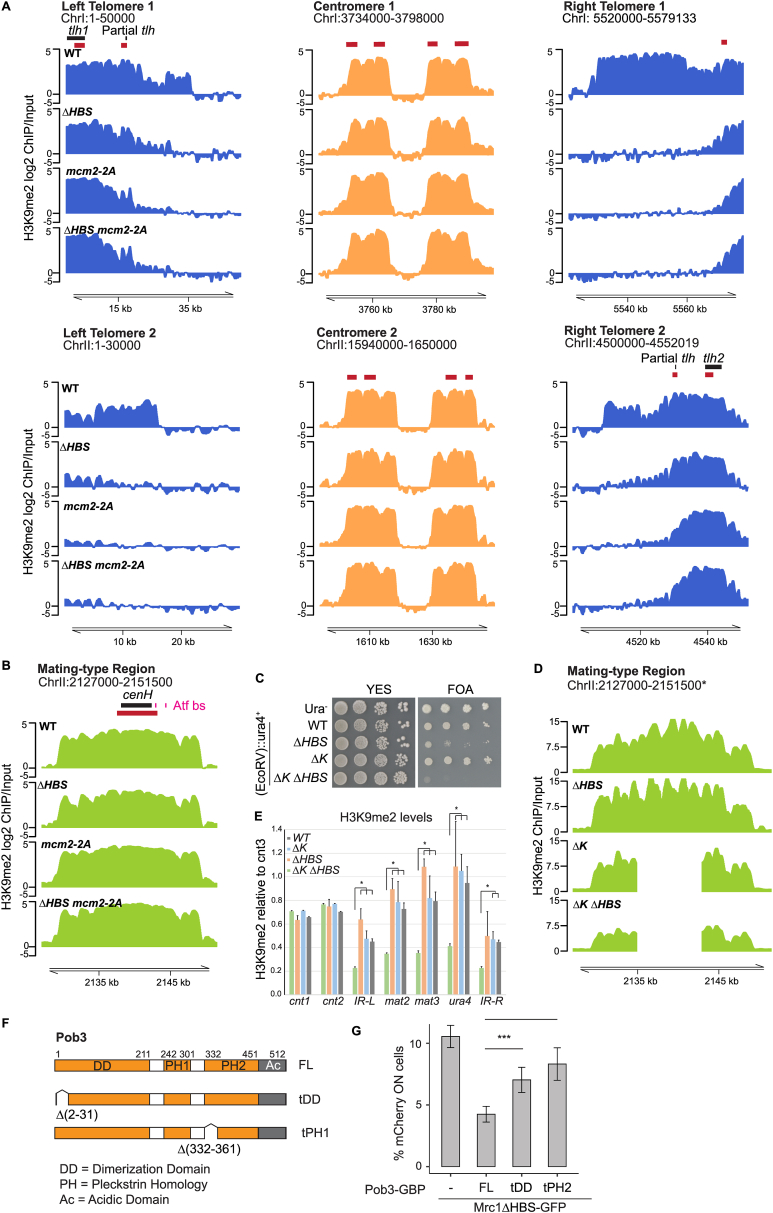
Heterochromatin loss away from nucleation sites in Mrc1 and Mcm2 mutants, related to Figure 3 (A) H3K9me2 occupancy at Chromosome 1 (top) and Chromosome 2 (bottom) in histone-recycling mutants. siRNAs are depicted as red blocks across Tel1L/Tel2L (left), centromeres (middle), or Tel1R/Tel2R (right). (B) H3K9me2 occupancy at the mating-type region indicating siRNAs originating from *cenH* depicted in red and the two Atf1-binding sites in magenta. (C) Ten-fold serial dilutions of cell suspensions were spotted onto the indicated media. Growth on the toxigenic substrate FOA reflects *ura4*^*+*^ repression. (D) H3K9me2 occupancy at the *h*^90^ and ΔK mating-type regions. (E) Quantification of H3K9me2 occupancy at indicated regions within the mating-type region normalized to *cnt3* [unpaired Student’s t test used]. (F) Schematic diagram of Pob3 with functional domains annotated and deletion mutations indicated. (G) Proportion of cells expressing *mCherry* in cells co-expressing Pob3-GBP (full-length and mutants) and Mrc1ΔHBS-GFP (mean ± SD, n = 6) [ANOVA, F = 41.6, P = 8.7 × 10^−9^].

The *mrc1*Δ*HBS* and *mcm2-2A* mutants showed a significant loss of H3K9me2 in sub-telomeric regions (Figure 3G), as mapped by chromatin immunoprecipitation sequencing (ChIP-seq). However, H3K9me2 was unaltered in zones of RNAi activity and at other nucleation elements, including centromeric repeats and sub-telomeric *tlh* genes^78^^,^^79^ (Figures 3G–3I, S3A, and S3B). This suggested that strong establishment signals mask roles of Mrc1 in heterochromatin maintenance. To test this, we used a *ΔK* strain in which the *cenH* RNAi nucleation site is deleted,^70^ containing also an *(EcoRV)::ura4*^*+*^ reporter. Strong growth on 5-fluoroorotic acid (FOA) plates indicated that heterochromatin can be stably maintained in the absence of the K region (Figure S3C), as reported.^69^^,^^70^ However, introduction of the *mrc1ΔHBS* mutation generated strong sensitivity to FOA and partial loss of H3K9me2 (Figures S3D and S3E). Residual H3K9me2 in the double mutant may stem from the remaining silencing elements present in the *ΔK* mating-type region.^32^^,^^33^^,^^35^^,^^71^^,^^77^^,^^80^ Collectively, this argues that defects in *cis*-based inheritance in Mrc1 and Mcm2 mutants challenge heterochromatin silencing mainly at sites lacking elements that drive establishment of silencing in each cell cycle.

Considering our observations, we speculated that the HBS domain could be involved in histone recycling. We therefore tested if it could be bypassed by artificially recruiting a known histone chaperone to the replisome. We tethered Pob3 to Mrc1ΔHBS using green fluorescent protein (GFP) and GFP-binding protein (GBP)^81^ (Figure 3J). An Mrc1ΔHBS-GFP fusion protein was expressed from the endogenous *mrc1* locus, and Pob3 fused with GBP was expressed ectopically in *pob3*^*+*^ strains harboring *(EcoRV)*:*:mCherry* at the mating-type region. Pob3-GBP significantly alleviated the silencing defect in the Mrc1ΔHBS-GFP strain (Figure 3K), and silencing defective Pob3 mutants carrying deletions in the dimerization domain or the pleckstrin homology domain^82^ impaired suppression (Figures S3F and S3G). This supported the idea that the Mrc1 HBS domain promotes histone chaperone activity at the replication fork.

### Mrc1 functions together with Mcm2 in recycling of parental histones to the lagging strand

To test a role of Mrc1 in histone recycling directly, we monitored the distribution of parental and new histone H3-H4 between sister chromatids by sister chromatids after replication by DNA sequencing (SCAR-seq).^49^^,^^83^ This technique can track the segregation of parental histones to the leading and lagging strands.

**Figure 4:**
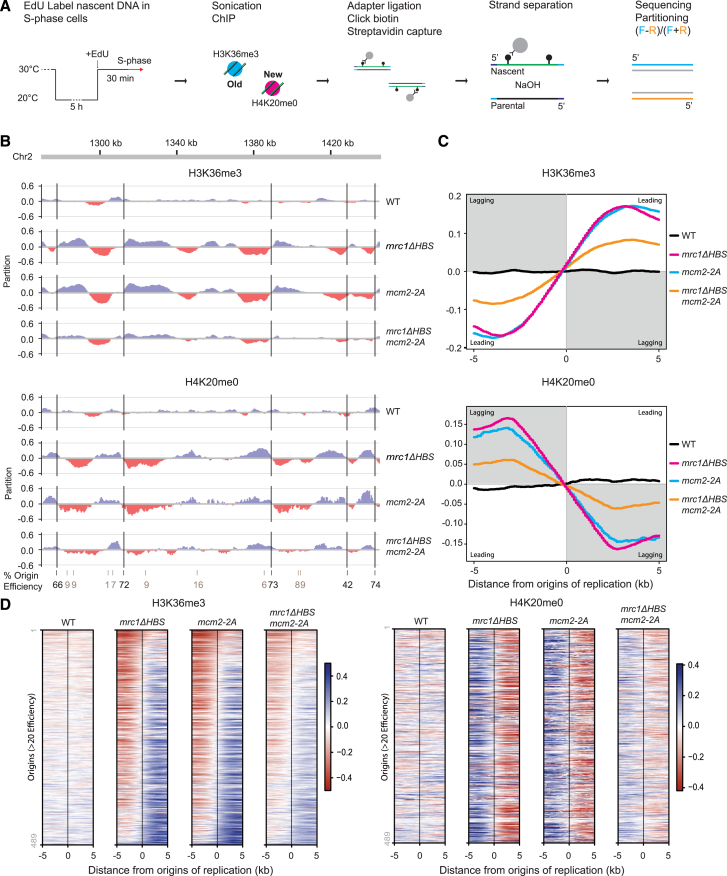
Mrc1 cooperates with Mcm2 in recycling of parental histones to the lagging strand (A) Workflow of xSCAR-seq in fission yeast. (B) Partition of H3K36me3 (top) and H4K20me0 (bottom) at a genomic region. Replication origin centers are depicted as black lines with their respective firing efficiency score. (C) Average partitioning score across replication initiation centers (with score >20) for parental (H3K36me3, top) and newly synthesized (H4K20me0, bottom) histones. (D) Heatmap representing the partitioning score across all replication origin centers (score >20) for H3K36me3 (left) and H4K20me0 (right). Each row represents the partition score of xSCAR-seq sequence reads at one origin. Average of two independent replicates. See also Figure S4.

**Figure S4:**
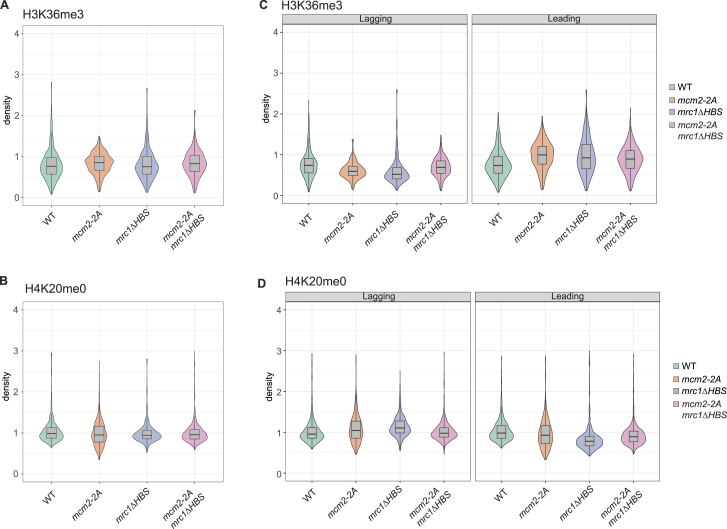
SCAR-seq density analysis reveals no overall loss or gain of parental and new histones in recycling mutants, related to Figure 4 (A and B) Density of parental histones (H3K36me3) and new histones (H4K20me0) on total replicated DNA (unstranded SCAR-seq/EdU Input) in ±2.5 kb bins centered on origins of replication. (C- and D) Density of parental histones (H3K36me3) and new histones (H4K20me0) on the leading and lagging strand (stranded SCAR-seq/EdU Input) in ±2.5 kb bins centered on origins of replication.

To establish SCAR-seq in fission yeast, we used histone H3K36me3 to track parental histones as we had done previously in mammalian cells.^49^ H3K36me3 is deposited during transcription and is not present on new histones.^84^^,^^85^ In parallel, we tracked newly synthesized histones using histone H4K20me0,^58^^,^^86^ as H4K20 is progressively methylated post nucleosome-assembly along the cell cycle.^84^^,^^87^ In fission yeast, S phase promptly follows M phase. We thus used the *nda3-KM311* allele encoding a cold-sensitive tubulin^88^ for cell-cycle synchronization in M phase at 20°C and released the cells at 30° C in the presence of 5-ethynyl-2'-deoxyuridine (EdU) to label the newly synthetized DNA (Figure 4A). For SCAR-seq, we combined ChIP for H3K36me3 and H4K20me0 with purification of EdU-labeled DNA, strand separation, and stranded sequencing.^49^ The partition was calculated from the ratio of forward and reverse reads (Figure 4A) and interrogated with respect to replication fork directionality from known origins of replication.^89^ *mcm2-2A* mutants showed highly asymmetric distribution of parental histones toward the leading strand (H3K36me3, Figures 4B–4D), and this was mirrored by enrichment of new histones on the lagging strand (H4K20me0, Figures 4B–4D), as reported in mammalian cells.^49^ Remarkably, the *mrc1*Δ*HBS* mutant replicated this histone-segregation defect and was virtually indistinguishable from the *mcm2-2A* mutant (Figures 4B–4D). This explains the defect in heterochromatin maintenance in these mutants and the asymmetric inheritance of derepression. In the double *mrc1*Δ*HBS mcm2-2A* mutant, the skew of parental histones toward the leading strand was less dramatic than in the single mutants (Figures 4B–4D). This lower degree of asymmetry could stem from either more efficient lagging-strand recycling or parental histone loss from the leading strand. Estimating histone-recycling efficiency by calculating the ratio of SCAR-seq signal over EdU inputs at initiation sites,^62^ stranded or unstranded, supported that lagging-strand recycling is partially rescued in the double mutant (Figure S4). This is consistent with suppression of the silencing defect in the *mrc1*Δ*HBS mcm2-2A* double mutant (Figure 3F). We concluded that, like Mcm2, Mrc1 facilitates transfer of parental histones to the lagging strand to ensure balanced segregation of histones in WT cells.

### Mrc1 coordinates parental histone inheritance within the replisome

**Figure 5:**
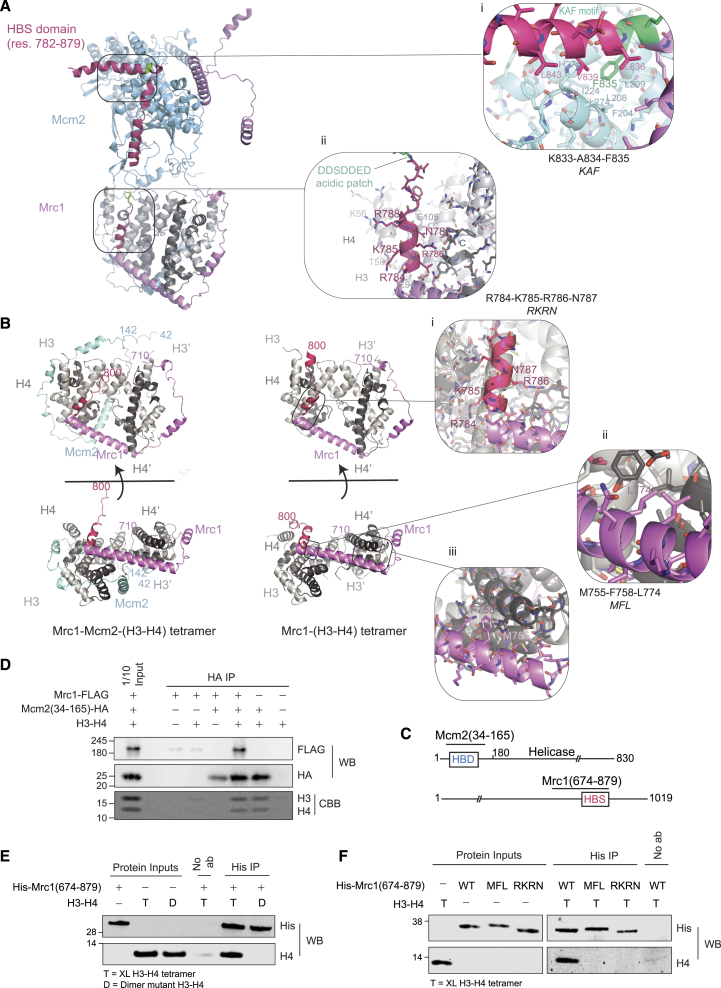
Mrc1 chaperones H3-H4 tetramers in a manner compatible with Mcm2 co-chaperoning (A) AF^90^^,^^91^ prediction of a complex comprising full-length *S. pombe* Mrc1 (pink) and Mcm2 (light blue) bound to a histone H3-H4 tetramer (gray). Histone tails and unstructured Mrc1 residues predicted with low confidence (residues 1–381, 475–710, 800–804, and 856–1,020) are not depicted for clarity. Closeups: (1) interaction of the Mrc1 KAF motif with a hydrophobic groove in Mcm2 and (2) interaction of the N-terminal alpha helix of the Mrc1 HBS domain with charged aa in histone H3 αN and α2 helices and histone H4 C terminus. (B) AF predictions of Mrc1 histone binding via the HBD, including beginning of the HBS domain (dark pink) and upstream region (710–800). Interaction between Mrc1 (pink) and the histones (gray) remains unchanged in the presence of Mcm2 (light blue). Closeups highlight residues subjected to mutational analysis. (C) Mcm2(34–165) and Mrc1(674–879) polypeptides used for pull-downs. (D) Pull-downs of full-length Mrc1-FLAG and H3-H4 with HA-Mcm2 HBD (34–165). Pulled-down proteins were detected by western blot (WB) or Coomassie staining (CBB). MW in KDa. (E) Pull-downs of H3-H4 dimers or tetramers with His_6_-Mrc1(674–879). (F) Pull-downs of H3-H4 tetramers with His_6_-Mrc1(674–879) WT and indicated mutants. See also Figure S5 and S6.

**Figure S5:**
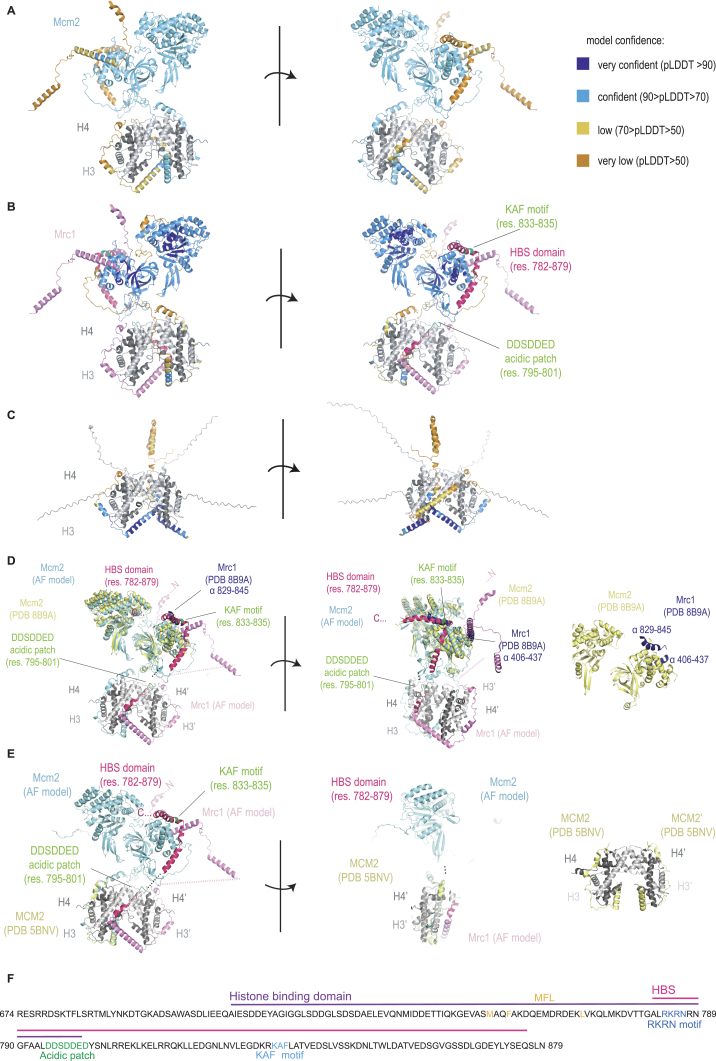
AlphaFold predicts an Mrc1-Mcm2 co-chaperone complex consistent with the Mcm2 HBD crystal structure and the Mcm2 cryo-EM structure at the replisome, related to Figure 5 (A and B) AF^90^^,^^91^ predictions of complexes comprising full-length Mrc1 and Mcm2 bound to a histone H3-H4 tetramer with Mrc1 (A) and Mcm2 (B) colored according to AF pLDDT (predicted local distance difference test) score to indicate prediction confidence. (C) AF predictions of a complex comprising full-length Mrc1 bound to an H3-H4 tetramer with Mrc1 colored according to AF pLDDT score. (D) The predicted interaction between Mcm2 (light blue) and Mrc1 (pink) mediated by Mrc1 helices (residues 829–845 and 406–437) is in agreement with Mcm2 (yellow)/Mrc1 (dark blue) positions previously experimentally determined by cryo-EM in the context of the replisome (PDB 8B9A).^4^ (E) The predicted interaction of the N-terminal domain of Mcm2 (light blue) with a histone H3-H4 tetramer is in agreement with an available crystal structure (yellow, PDB 5BNV^51^). (F) Amino acid sequence of the Mrc1(674–879) fragment used in *in vitro* pull downs with relevant domains and residues highlighted.

**Figure S6:**
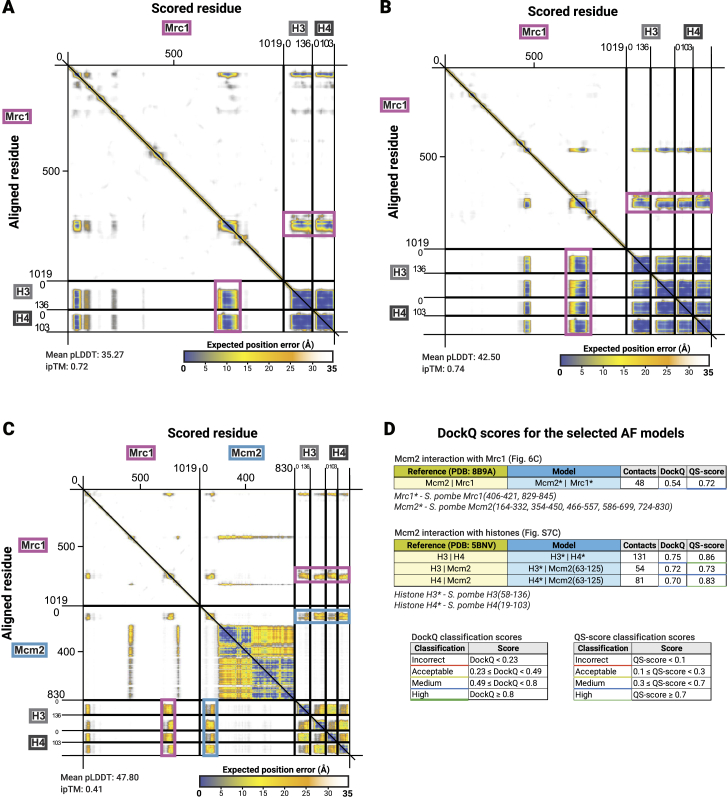
PAE plots for AF best scoring models, related to Figure 5 (A) Mrc1 with histone dimer, (B) Mrc1 with histone tetramer, and (C) Mrc1 and Mcm2 with histone tetramer. The predicted interaction interfaces of Mrc1 and Mcm2 with histones are highlighted in pink and light blue, respectively. Predicted aligned error (PAE) plots^90^^,^^91^ were visualized by ChimeraX 1.7^127^. Tables in (D) show DockQ^133^ and QS-score^134^ validation scores for Mcm2 and Mrc1 (Figure 6C), and Mcm2 and H3/H4 histone (Figure S5C) interaction interface in Mrc1-Mcm2-H3/H4 tetramer AF model compared to selected experimental protein structures (PDB: 8B9A^4^ and 5BNV,^51^ respectively—see STAR Methods).

We made several predictions with AlphaFold multimer (AF)^90^^,^^91^ toward understanding how Mrc1 could support histone recycling. We considered that Mrc1 might bind histones both alone and together with Mcm2, as many dynamic transactions involving histones are mediated by co-chaperoning events where proteins concomitantly engage with and guide histones to their destination.^92^ FACT and Asf1 can co-chaperone histone H3-H4 with replisome components MCM2^48^^,^^51^^,^^52^^,^^60^^,^^62^^,^^93^ and Polα (FACT only).^59^ This prompted the model that soluble chaperones transport histones across the replisome using embedded histone-binding interfaces as steppingstones.^1^^,^^48^ No co-chaperone relationships have been identified between replisome factors, although this could provide highly controlled movement of histones across the replisome. Our analysis predicted that Mrc1 can bind an H3-H4 tetramer alone or together with Mcm2 (Figures 5B, S5, and S6) in a manner where the H3-H4 tetramer maintains a conformation similar to that in the nucleosome.^94^ Notably, the predicted histone-binding mode for Mrc1 is highly similar with and without Mcm2 (Figures 5B and S5F), involving residues 710–790 that include the beginning of the HBS domain (aa 782–879) (Figure 5B). This region wraps around one dimer and continues in a long α-helix that bridges the two H3-H4 dimers. This binding mode is compatible with Mcm2 wrapping around the other H3-H4 dimer, analogous to the crystal structure of mammalian MCM2 with H3-H4.^51^^,^^60^ Together, Mrc1 and Mcm2 thus envelop the tetramer.

The Mrc1 HBS domain is predicted to facilitate co-chaperoning by tethering the Mrc1 HBD to Mcm2 (Figure 5A): the KAF motif makes direct contact with a hydrophobic groove formed by conserved Mcm2 residues F204, L208, L274, and Y276 (Figure 5A, inlet i), while the DSE motif acts as the central flexible connective linker between these two entities (Figure 5A, inlet ii). This bridging function of the HBS domain is consistent with XL-MS^2^ and cryo-EM data^4^ and was further supported by superposition of our Mcm2-(H3-H4)_2_-Mrc1 co-chaperone model onto the cryo-EM structure of the *S. cerevisiae* replisome, which includes the specific region where the KAF motif is located (Figure S5D). Cryo-EM data^4^ also validates a second interaction of Mrc1 with Mcm2, predicted in our co-chaperone model. This interaction is mediated by aa 406–437 in the NTHBS domain, likely explaining why this region is important for silencing (Figure S5D). On the H3-H4 tetramer side, the conserved Mrc1 HBS domain residues R784, K785, R786, and N787 are predicted to contact the C terminus of H4 as well as the α2-helix and L1-linker of H3 in the dimer, also bound by Mcm2 (Figure 5B, inlet i). Deletion of the HBS domain would remove a critical region that both binds H3-H4 and bridges it to Mcm2, thereby positioning the two chaperone domains correctly for co-chaperoning H3-H4 tetramers on their path to the lagging strand. This co-chaperone model thus immediately explains the similar phenotype and interaction of the *mcm2-2A* and *mrc1ΔHBS* mutants.

**Figure S7:**
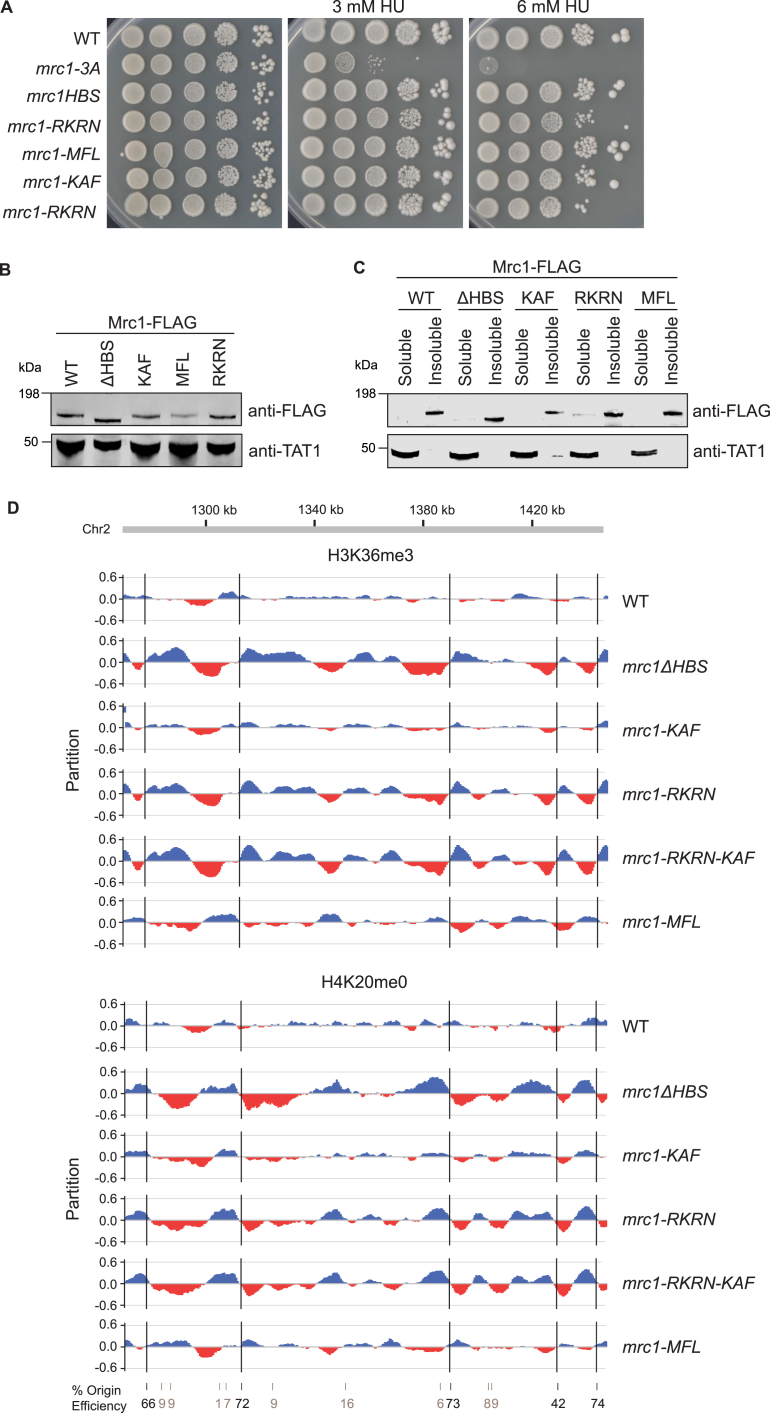
Characterization of Mrc1 histone-recycling mutants, related to Figure 6 (A) Ten-fold serial dilutions of cell suspensions were spotted on HU-containing medium to estimate checkpoint proficiency. (B) Immunoblot of Mrc1-FLAG and tubulin from whole cell extracts of S. pombe mrc1 mutants. (C) Immunoblot of Mrc1-Flag upon chromatin fractionation of S. pombe mrc1 mutants performed as in Shimmoto et al. (2009).^136^ Tubulin serves as soluble control. (D) Partition of H3K36me3 (top) and H4K20me0 (bottom) at a genomic region. Replication origin centers are depicted as black lines with their respective firing efficiency score.

**Figure 6:**
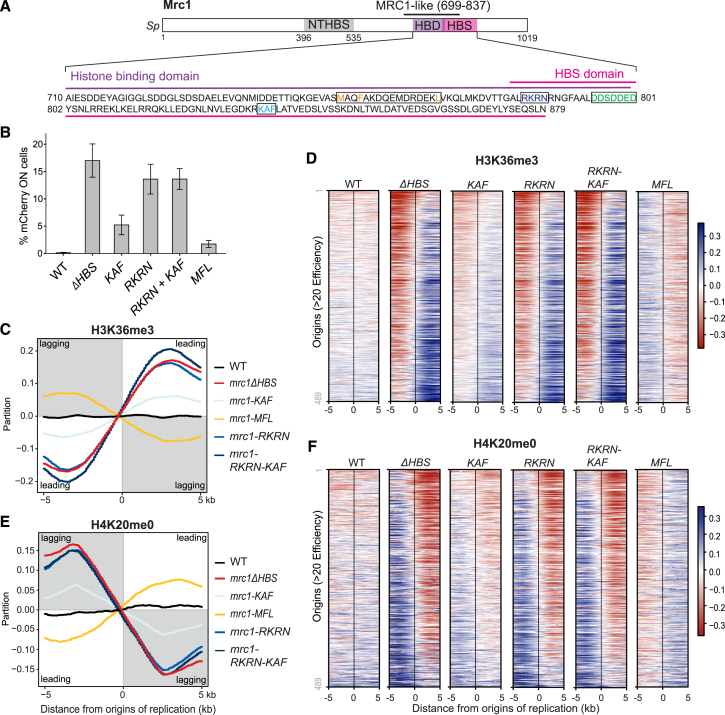
Mrc1 coordinates parental histone inheritance within the replisome for heterochromatin maintenance (A) Overview of the Mrc1-HBD (710–800; based on Figure 5B) and the HBS (782–879) domains. Colored residues were mutated in this study. (B) Cells expressing *mCherry* (*n* = 6). WT, *mrc1*Δ*HBS*, and *mrc1-KAF* values are included for comparison from Figure 2B. Data are represented as mean ± SD. (C and E) Average partitioning score across replication initiation centers (with score >20) for parental (H3K36me3) histones (C) and newly synthesized (H4K20me0) histones (E). (D and F) Heatmap representing the partitioning score across all replication origin centers (score >20) for H3K36me3 (D) and H4K20me0 (F). See also Figure S7.

To test the AlphaFold prediction that Mrc1 and Mcm2 concomitantly bind H3-H4, we purified full-length Mrc1 and an N-terminal fragment of Mcm2(34–165) containing the HBD but not the Mrc1 binding site (Figure 5C). *In vitro* pull-downs using Mcm2 HBD as bait revealed that while Mrc1 did not interact directly with the Mcm2 HBD, Mrc1 was pulled down by the Mcm2 HBD in the presence of H3-H4 (Figure 5D). This argues that Mrc1 binds histone H3-H4 in a manner that is compatible with co-chaperoning with Mcm2. To further investigate the histone-binding capacity of Mrc1, we purified Mrc1(674–879) containing both the HBS domain and the upstream domain predicted to interact with histones (Figures 5C and S5F). We performed *in vitro* pull-downs with constitutive H3-H4 dimers and cross-linked H3-H4 tetramers^95^ and found that Mrc1 binds H3-H4 histones with a marked preference for tetramers over dimers (Figure 5E). We then pinpointed Mrc1 amino acids inside and outside the HBS region predicted to mediate interaction with H3-H4, and we designed an RKRN mutant (R784A, K785A, R786A, and N787A) to disrupt interactions with the H3 α2-helix and the C-terminal of H4 and an MFL mutant (M755A, F758A, and L774A) to target key residues predicted to anchor the long Mrc1 helix to the two H3-H4 dimers (Figure 5B, inlets ii and iii). The MFL mutant was recently found to disrupt histone binding by the Moazed laboratory (D. Moazed, personal communication). In our hands, both mutants exhibited a reduced interaction with H3-H4 *in vitro* (Figure 5F). *In vivo*, the two mutants were proficient for the replication checkpoint, and the proteins were recruited to chromatin similar to WT Mrc1 (Figures S7A–S7C), but they behaved differently with respect to silencing and histone recycling (Figures 6A–6F). RKRN mutations, a change predicted to affect the connector function of the HBS domain by weakening the anchor point of Mrc1 to the H3-H4 tetramer, gave rise to a pronounced silencing defect comparable to deletion of the full HBS domain (Figure 6B). In agreement, SCAR-seq showed a strongly biased segregation of parental histones toward the leading strand, recapitulating the full HBS deletion (Figures 6C, 6D, and S7D). MFL mutations, predicted to affect the anchoring of the two H3-H4 dimers to Mrc1 (Figure 5B), also showed a silencing defect, although it was less prominent than RKRN (Figure 6B). Surprisingly, this mutant gave rise to an opposite segregation bias of more modest amplitude, where old histones segregated preferentially to the lagging strand (Figures 6C, 6D, and S7D). The two mutants thus separate functions of Mrc1 in the recycling of parental histones to the lagging strand, for which RKRN is required, and the leading strand, for which MFL is required.

We went on to test whether the Mrc1 KAF and RKRN motifs cooperate as predicted by our model where they would function as anchor points to co-chaperone H3-H4 with Mcm2. As predicted, mutations affecting the KAF and RKRN motifs were epistatic, both with respect to loss of silencing (Figure 6B) and histone inheritance defect (Figures 6C–6F), impairing lagging-strand segregation identically to the full HBS mutant.

Collectively, this identifies a histone H3-H4 chaperone functionality of Mrc1 involved in inheritance of parental histones across the replisome to both the leading and lagging strand, working with Mcm2 in the latter pathway. This histone-recycling function, including both the HBD and the HBS connector and Mcm2-binding regions, falls within the conserved MRC1-like domain (Figure 6A).

### CLASPIN^Mrc1^ regulates histone recycling in mammalian cells

**Figure 7:**
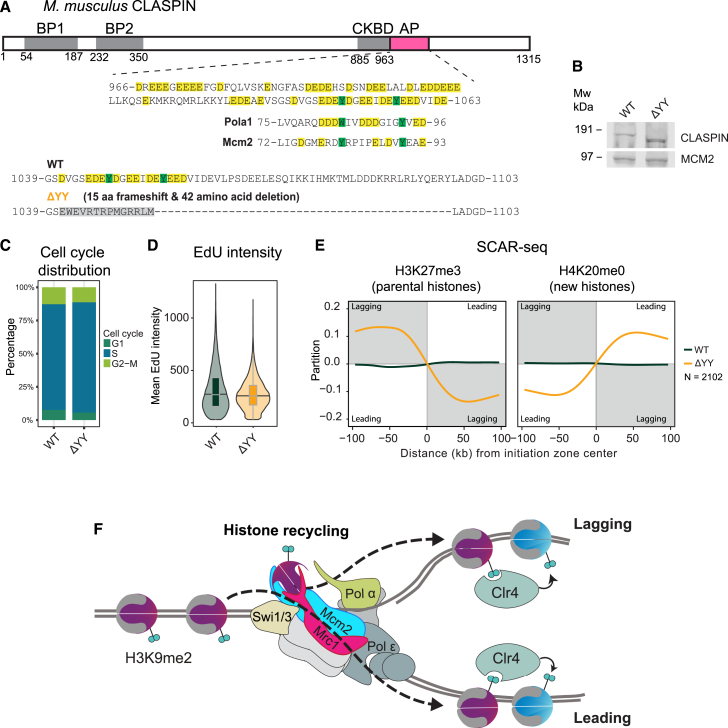
Histone recycling function of Mrc1 is conserved in mammalian cells (A) (top) Mouse CLASPIN with annotated domains (BP1 and BP2: basic patch 1 and 2, CKBD: Chk1 binding domain, AP: acidic patch) and alignment with Polα and Mcm2 HBDs. (Bottom) overview of mutation in CLASPINΔYY. (B) WB analysis of WT and CLASPIN mutant mESCs. (C) Cell-cycle distribution based on mean EdU intensity and total DAPI intensity of WT and CLASPIN mutant mESCs. (D) High-content microscopy of mean EdU intensity in WT and CLASPIN mutant mESCs. (E) Average SCAR-seq profile of H3K27me3 and H4K20me0 in WT and CLASPIN mutant mESCs. (C–E) *n* = 2 biological replicates. (F) Model: Mrc1 acts as a central coordinator of histone-based inheritance though its ability to bind and transfer H3-H4 tetramers to both leading and lagging strands, with the latter involving joint histone binding with Mcm2 to facilitate transfer to Polα and the lagging strand.

Given the high functional conservation of yeast and mammalian recycling factors, we tested the mouse homolog CLASPIN for a role in histone recycling. While the sequence conservation of CLASPIN and Mrc1 is limited, previous work has suggested that an extended acidic patch region in CLASPIN has functional similarity to the HBS domain of Mrc1 with respect to replication initiation.^96^ In this region, we noted a site reminiscent of the histone-binding motifs in POLA1 and MCM2,^51^^,^^52^^,^^59^ where two aromatic residues 8–10 aa apart are embedded in an acidic region (Figure 7A). We performed genome editing in mESCs and obtained a deletion mutant with a short frameshift, removing the YY motif and part of the downstream acidic patch (ΔYY) and giving rise to a smaller protein expressed at WT levels (Figures 7A and 7B). The mutant cells were viable with unaltered cell-cycle distribution and normal rates of DNA replication (Figures 7C and 7D), indicating that essential functions of CLASPIN in replication initiation and fork progression are unaffected.^5^^,^^10^^,^^96^ SCAR-seq for parental and new histones using H3K27me3 and H4K20me0,^55^^,^^58^ respectively, revealed a strong lagging-strand bias for parental histones, mirrored by a skew of new histones to the leading strand (Figure 7E). This argues that CLASPIN is required for recycling of parental H3-H4 to the leading strand and that a role of CLASPIN^Mrc1^ in histone recycling is conserved between yeast and mammals.

## Discussion

Here we uncover a central function of Mrc1 in transmitting parental histones symmetrically to both daughter strands and show that this function is necessary for the faithful inheritance of silent heterochromatic domains upon passage through S phase. Our findings both identify a coordinator function in histone recycling at the replisome and support the important concept that asymmetric histone segregation during DNA replication can lead to the clonal loss of epigenetic memory. The salient lines of evidence supporting this conclusion are (1) *mrc1* mutants show asymmetric clonal loss of H3K9me2 heterochromatic silencing similar to *mcm2-2A* mutants, (2) *mrc1ΔHBS* mimics the *mcm2-2A* mutant functionally and phenotypically, including a similar strong lagging-strand-recycling defect, and the double mutant phenocopies the single mutants with a trend toward suppression, (3) AlphaFold predictions and *in vivo* interaction assays support that Mrc1 could function with Mcm2 by co-chaperoning histone H3-H4 tetramers and in histone transfer to the lagging strand, (4) Mrc1 binds histone H3-H4 tetramers, not dimers, in a manner relying on the predicted interactions, (5) an Mrc1 histone-binding mutant in the HBS connector domain recapitulates the full HBS deletion and is epistatic with mutation of the Mcm2 tethering site, (6) two Mrc1 histone-binding mutants—inside and outside the HBS domain—show opposite strand biases in parental histone segregation, placing Mrc1 in both leading- and lagging-strand recycling, and finally (7) silencing defects correlate tightly with the degree of asymmetric recycling across all Mrc1 mutants, arguing that correct balanced segregation of parental histone is required for epigenetic inheritance.

### Mrc1, an H3-H4 chaperone with possibility to toggle histones between leading and lagging strand

We identified an HBD in Mrc1 with preference for histone H3-H4 tetramers over dimers and validated two key interaction sites from AlphaFold models of Mrc1 co-folded with H3-H4 tetramers, alone and with Mcm2. In these models, a long α helix of Mrc1 bridges the two H3-H4 dimers. Mrc1 further clamps onto the H3 α2 helix of one dimer and wraps around the other dimer, which could stabilize the tetramer on its path across the replisome. This is diffferent from other replisome-integrated histone chaperones, as MCM2 and POLE3/E4 can bind both H3-H4 tetramers and dimers^51^^,^^60^^,^^97^ (this remains to be tested for POLA1). Furthermore, Mrc1 bridges the H3-H4 tetramer in an unusual manner^92^ on the opposite side of the dyad, which in principle makes the dyad available for DNA binding and deposition.

The Mrc1-binding mode for H3-H4 tetramers is compatible with Mcm2 wrapping around the other H3-H4 dimer, with its well-established HBD in a conformation similar to the crystal structure of MCM2 with H3-H4.^51^^,^^60^ Consistent with this, a histone H3-H4-dependent complex between Mrc1 and the Mcm2 HBD can form *in vitro*. The location of Mrc1 (*S. cerevisiae* or human CLASPIN) has only been sketchily mapped at replisomes. Yet, structural and XL-MS data^2^^,^^4^ show the close proximity of the highly conserved KAF motif in the Mrc1 HBS domain with the point where the Mcm2 HBD branches off from the helicase. The predicted proximity, along with their compatible histone-binding modes (this work; Huang et al. [2015]^51^ and Richet et al. [2015]^60^), argues for a functional cooperation between Mrc1 and Mcm2 through co-chaperoning in histone transfer to Polα^53^ for lagging-strand deposition possibly via DNA polymerase δ.^98^^,^^99^ This model is supported by genetics placing the Mrc1 HBS domain and Mcm2 HBD in the same pathway as well as by molecular and functional analyses: *mrc1ΔHBS* and *mcm2-2A* mutants show clonal, asymmetric loss of silencing and a similar strong defect in lagging-strand histone recycling. This phenotype is mimicked by the RKRN mutant, predicted to lose its grip on the histone dimer also engaged by Mcm2 in the co-chaperone complex, and partly reproduced by the KAF mutant targeting the Mcm2 tethering site. An MCM2 mutation in this site was also recently identified in a loss-of-silencing screen.^54^ Collectively, this places Mcm2 and the Mrc1 HBS domain in the same lagging-strand histone-recycling pathway. Accordingly, the double mutant (*mrc1ΔHBS mcm2-2A*) also shows a lagging-strand-recycling defect and loss of silencing, although less so than the *mrc1ΔHBS* mutant alone. In our eyes, the most parsimonious explanation is that trapping of histone H3-H4 by the strong-affinity binding site on Mcm2^51^^,^^52^^,^^60^ is one of the problems in the *mrc1ΔHBS* mutant, which would be consistent with a requirement for co-chaperoning in histone transfer to Polα. In the absence of Mcm2 histone binding, Mrc1ΔHBS might maintain a degree of histone binding (see next paragraph) that along with unhooking from Mcm2 would enable histone transfer to the lagging strand, although inefficiently. However, other scenarios are possible especially as FACT could also be involved in Mrc1-and Mcm2-mediated histone recycling.^52^^,^^62^^,^^63^^,^^64^

Surprisingly, the functional analysis of Mrc1 histone-binding mutants enabled us to separate functions of Mrc1 in the lagging- and leading-strand recycling pathways. This suggests that Mrc1 is in a coordinator position with the ability to toggle between the two pathways. This could involve separate histone handover reactions with different requirements for how Mrc1 engages histones, i.e., Mcm2 co-chaperoning on the lagging strand. Future exploration of this model and a potential “pathway switch” would benefit from capturing histones in transfer in structural studies of the replisome.

We identify a function of CLASPIN in recycling parental H3-H4 to the leading strand. Future work is required to address if CLASPIN has histone-binding functionality and a dual function in leading- and lagging-strand recycling like Mrc1. We note that the CLASPIN^Mrc1^-MCM2 interaction was determined by cryo-EM of the human replisome,^3^ suggesting that the functional relationship with MCM2 is also conserved.

Taking current literature into account, we envision the following sequence of events. FACT interacting with the Tof1/Swi1 subunit of the FPC would bind to partially unwrapped parental nucleosomes as they are encountered by the progressing replisome.^61^ In coordination with FACT and Tof1/Swi1, histone H3-H4 tetramers would be captured by Mcm2 as structural work has elegantly demonstrated.^51^^,^^60^^,^^63^^,^^64^ Mrc1 would, through co-chaperoning with Mcm2, facilitate their subsequent transfer to Ctf4-Polα and Pol δ for nucleosome assembly on the lagging strand.^49^^,^^50^^,^^53^^,^^98^^,^^99^ A separate handover event, possibly with direct delivery from FACT, would enable Mrc1 to transmit parental H3-H4 tetramers to Dbf3/4 and the leading strand. We favor the idea of histone co-chaperoning between core replisome factors acting in concert with soluble chaperones. This may explain how Mrc1 coordinates distribution of histones to both lagging- and leading-strand pathways to ensure transmission of epigenetic information to both daughter cells (Figure 7F). Furthermore, we speculate that this function of Mrc1 could be integrated with its other activities like fork protection and fork progression.

### Importance of symmetric histone transfer in nucleosome-based epigenetic inheritance

Our data showcase how histone recycling contributes to epigenetic inheritance in natural chromatin contexts. Histone-based propagation proved critical at a distance from silencing nucleation sites or when nucleation was weakened. Thus, heterochromatin was robustly maintained at genomic locations close to RNAi activity where it is boosted by direct contacts between RNAi and CLRC complexes rather than by chromodomain-mediated feedback.^39^^,^^100^ RNAi relies on transcription which is stimulated by each passage through S phase,^30^^,^^31^ a fast-acting silencing process.^66^ In the mating-type region with multiple cooperating nucleation sites, a large heterochromatic domain remained in the Mcm2 and Mrc1 mutants, but it was destabilized when RNAi was inactivated by deleting the *cenH* element or when the *REII* element was removed. This corroborates and extends observations in mouse ESCs carrying the MCM2-2A mutation, where asymmetric histone segregation leads to loss of H3K9me3 and desilencing at some but not all repeats, consistent with differential dependence on read-write propagation linked to the strength of establishment signals in a given region.^53^^,^^55^

Our observations thus lead to a simple model for the loss of heterochromatin in Mrc1 mutants where the occasional loss of a critical number of parental histones in a heterochromatic domain would result in the loss of epigenetic memory. This model relies on the fact that positional information is maintained during recycling of parental histones^101^^,^^102^^,^^103^ and that a critical threshold of modified nucleosomes is necessary for maintenance by read/write activities in fission yeast.^104^ Loss-of-silencing events might be expected to be relatively rare, as we observed in FPC mutants through single-cell assays. In budding yeast, experimentally reducing the number of nucleosomes at the silenced *HMR* locus failed to destabilize silenced chromatin as might be predicted by histone-based inheritance models.^105^ However, in other cases, programmed long-range biases in parental histone segregation have been proposed to mediate asymmetric cell divisions of *Drosophila* germline stem cells.^106^^,^^107^^,^^108^ Our observations linking asymmetric recycling with clonal inheritance of the derepressed state provide strong support for the notion that *cis*-based inheritance of histones mediates epigenetic memory.

### Limitations of the study

The Mrc1 RKRN motif, part of the HBS and the HBD, is one of three putative NLS motifs in Mrc1.^109^ A contribution of this motif to nuclear import is therefore also plausible. However, the Mrc1 RNRN and ΔHBS mutants are checkpoint proficient (Figure S7A), and both proteins bind chromatin-like WT Mrc1 (Figure S7C). In addition, the Mrc1ΔHBS mutant protein (lacking the RKRN motif) is efficiently recruited to replication forks.^22^ Together, this provides evidence that a role of RKRN in nuclear import is separate from functions in histone recycling. Our study provides evidence of intra-replisome co-chaperone in histone recycling and an entry point to understand how histones might be toggled between the leading- and lagging-strand pathways. A full understanding of the paths that histone (H3-H4)_2_ takes across the replisome and hand-over mechanisms will require comprehensive functional and structural investigations of replisome-integrated histone-binding activities and how they engage with mobile chaperones like FACT and ASF1.

## STAR★Methods

### Key resources table

**Table:** 

REAGENT or RESOURCE	SOURCE	IDENTIFIER
**Antibodies**		

H4K20me0 (rabbit)	Abcam	Cat no. ab227804
H3K36me3 (rabbit)	Abcam	Cat no. ab9050; RRID: AB_306966
H3K9me2 (mouse)	Abcam	Cat no. ab1220; RRID: AB_449854
H3K27me3 (rabbit)	Cell Signaling	Cat no. 9733; RRID: AB_2616029
His (mouse)	Abcam	Cat no. ab18184; RRID: AB_444306
Flag-M2 (mouse)	Sigma-Aldrich	Cat no. F3165; RRID: AB_259529
TAT-1 (α-tubulin) (mouse)	Merck	Cat no. 00020911; RRID: AB_10013740
MCM2 (rabbit)	Cell Signaling	Cat no. 3619; RRID: AB_2142137
CLASPIN (rabbit)	Bethyl Laboratories	Cat. No A300-266A; RRID: AB_155895
CW680 anti-mouse	LI-COR	RRID AB_10953628
CW800 anti-rabbit	LI-COR	RRID AB_621843
BrdU (mouse)	MBL	Cat no. MI-11-3; RRID: AB_590678
Direct-Blot HRP anti-HA.11 Epitope Tag	BioLegend	Cat. 901520; RRID: AB_2749912
anti-FLAG M2 antibody horseradish peroxidase (HRP)	Sigma-Aldrich	Cat no. A8592; RRID: AB_439702

**Bacterial and virus strains**		

Rosetta (DE3) Competent cells	Novagen	Cat no. 70954

**Chemicals, peptides, and recombinant proteins**		

5-Fluoroorotic acid	US Biological	Cat no. F5050
DMEM	Gibco	Cat no. 31966
Fetal Calf Serum	GE Hyclone	Cat no. SV30160.03
Penicillin/Streptomycin	GIBCO	Cat no. 151400122
Non-essential amino acids	GIBCO	Cat no. 11140050
2-Mercaptoethanol	GIBCO	Cat no. 21985023
Leukemia Inhibitory Factor	Custom-made	N/A
Gelatin from bovine skin	Sigma	Cat no. G9391
Lipofectamine 3000	Thermo Fisher Scientific	Cat no. L3000015
DMSO	Sigma	Cat no. D2650
5-bromo-2′-deoxyuridine	Sigma-Aldrich	Cat no. B5002
Hydroxyurea (*S. pombe* BrdU-seq)	FUJIFILM Wako Pure Chemical Corporation	Cat no. 089-06651
5-ethynyl-2′-deoxyuridine (mESC SCAR-seq)	Invitrogen	Cat no. A10044
5-ethynyl-2′-deoxyuridine (*S. pombe* xSCAR-seq)	Jena Bioscience	Cat no. CLK-N001-500
Formaldehyde	Thermo Fisher	Cat no. 28908
Dynabeads Protein G (ChIP-seq)	Thermo Fisher	Cat no. 10004D
Dynabeads Protein A (xSCAR-seq)	Thermo Fisher	Cat no. 10002D
AMPure XP beads	Beckman Coulter	Cat no. A63881
Biotin-TEG-Azide	Berry & Associates	Cat no. BT1085
THPTA	Sigma	Cat no. 762342
Dynabeads MyOne Streptavidin T1	Thermo Fisher	Cat no. 65602
Dynabeads M-280 Sheep Anti-Rabbit IgG (SCAR-seq)	Thermo Fisher	Cat no. 11204D
ANTI-FLAG M2 Affinity Gel	Sigma-Aldrich	Cat no. A2220
3xFlag Eluting Peptide	ABGENT	Cat no. BP1013i
BMOE (bismaleimidoethane)	Thermo Fisher	Cat no. 22323

**Critical commercial assays**		

Click-iT EdU Kit	Thermo Fisher	Cat no. C10337
MinElute Reaction Cleanup Kit	QIAGEN	Cat no. 28204
KAPA Hyperprep Kit	Kappa Biosystems, Roche	Cat no. KK8504

**Deposited data**		

Raw and analyzed data	This paper	GEO: GSE241063
Western Blot Images	This paper	https://sid.erda.dk/sharelink/hkAiSCzwqV
Replication origins (*S. pombe*)	Daigaku et al.^89^	GEO: GSE62108
Replication origins (mESC)	Petryk et al.^49^	GEO: GSE117274

**Experimental models: Cell lines**		

Mouse: E14 ES cells (WT)	Laboratories of K. Helin and J. Brickman	RRID:CVCL_C320
Mouse ESC: CLASPIN_del1041-1098	This study	RRID:CVCL_C320

**Experimental models: Organisms/strains**		

*S. pombe strains*	This study	Supplementary Table S1

**Oligonucleotides**		

Oligonucleotides	This study	Supplementary Table S2
xGen UDI-UMI Adapters, 1–96, set	Integrated DNA Technologies	Cat no. 10005903

**Recombinant DNA**		

pSC20 (Mrc1)	This study	N/A
pSC21 (Mrc1ΔNTHBS)	This study	N/A
pAW2 (Mrc1-S353A-S797A)	This study	N/A
pSC67 (Mrc1-MFL)	This study	N/A
pSC43 (Mrc1-RKRN)	This study	N/A
pSC48 (Mrc1-RKRN-KAF)	This study	N/A
pSC13 (Mrc1-GFP-HA)	This study	N/A
pSC37 (Mrc1-DSE-GFP-HA)	This study	N/A
pSC38 (Mrc1-KAF-GFP-HA)	This study	N/A
pSC22 (Mrc1ΔHBS-GFP-HA)	This study	N/A
pSC26 (Pob3-GBP)	This study	N/A
pSC32 (Pob3Δ(2–33)-GBP)	This study	N/A
pSC33 (Pob3Δ(332–361)-GBP)	This study	N/A
pPA53 (Mcm2-2A)	This study	N/A
pCPR0021 (SUMO-HA-SpMcm2(34–165))	This study	N/A
pCPR0021 (6xHis-SpMrc1(674–879))	This study	N/A
pCPR0021 (6xHis-SpMrc1(674–879)-MFL)	This study	N/A
pCPR0021 (6xHis-SpMrc1(674–879)-RKRN)	This study	N/A

**Software and algorithms**		

Xcytoview v.1.1.11.0	ChemoMetec	N/A
Alphafold multimer	Evans et al.,^90^ Jumper et al.^91^	https://github.com/google-deepmind/alphafold
Pymol	Pymol team^110^	https://www.pymol.org/
ChimeraX	Meng et al.^111^	https://www.rbvi.ucsf.edu/chimerax/
Bowtie2 v2.4.2	Langmead and Salzberg^112^	https://github.com/BenLangmead/bowtie2
SAMtools v1.12	Danecek et al.^113^	http://www.htslib.org/
MACS2 v	Zhang et al.^114^	https://github.com/taoliu/MACS/tree/master/MACS2
IGV	Robinson et al.^115^	https://igv.org/
Galaxy pipeline	Galaxy team^116^	https://usegalaxy.org/
Deeptools v.3.5.1	Ramirez et al.^117^	https://deeptools.readthedocs.io/en/develop/
SeqPlots v.12.1	Stempor and Ahringer^118^	https://bioconductor.org/packages/release/bioc/html/seqplots.html
R and R Studio	R Project	https://www.r-project.org/
SCAR-Seq Analysis Pipeline	Wenger et al.^55^	https://github.com/anderssonlab/Wenger_et_al_2022

**Other**		

HisTrap affinity column	Cytiva	Cat no. 17525501
HiLoad 16/600 Superdex 200 Column	Cytiva	Cat no. 28989335
HiLoad 16/600 Superdex 75	Cytiva	Cat no. 28989333
Spectra/Por® 2 Dialysis Membrane	Spectrum Laboratories	Cat no. 11485849

### Resource availability

#### Lead contact

Further information and requests for resources and reagents should be directed to and will be fulfilled by the lead contact, Geneviève Thon (gen@bio.ku.dk).

#### Materials availability

Targeting constructs for genome editing and newly generated strains are available upon request and should be directed to lead contact, Geneviève Thon.

#### Data and code availability

ChIP/xSCAR-Seq data have been deposited at GEO under GSE241063 and are publicly available as of the date of publication. Accession numbers are listed in the key resources table. Alphafold predictions and raw data from western blot have been deposited at the Electronic Research Data Archive at the University of Copenhagen (https://sid.erda.dk/sharelink/hkAiSCzwqV).

This study did not generate original code. All computational approaches and software used are described in the STAR Methods and listed in the key resources table.

Any additional information required to reanalyze the data reported in this paper is available from the Lead contact upon request.

### Experimental model and study participant details

#### Model organisms

The experiments presented were conducted with the fission yeast *Schizosaccharomyces pombe* and with mouse ESCs derived from the male E14JU line with a 129/Ola background. *S. pombe* strains used were constructed by transformation^119^ or genetic crosses.^120^ Chromosomal DNA extraction was performed according to a published protocol.^121^ Strains are listed in Table S1. Mouse ESC mutant cell lines were generated by CRISPR-Cas9 mediated genome editing.

#### Growth conditions

*S. pombe* media (YES, EMM2, MSA) were prepared according to published protocols.^122^ YE (5 g/L yeast extract and 30 g/L glucose, a rich medium with low adenine concentration) was used to assay *(EcoRV)::ade6*^*+*^ expression and AA dropout medium lacking uracil or containing 5-Fluoorotic acid was used to assay *(XbaI)::ura4*^*+*^. YES containing 3 or 6mM hydroxyurea (HU) were used in assay checkpoint proficiency. To incorporate 5-Bromo-2′-deoxyuridine (BrdU) in replicating cells, *nda3-KM311* cells were incubated for 5 h at 20°C for synchronisation in M phase^123^ and released at 30°C in YES containing 200 μg/mL BrdU and 25 μM HU, added 30 min before release. After 1 h cells were harvested, flash-frozen, and stored at −80°C. To incorporate 5-Ethynyl-2′-deoxyuridine (EdU) in replicating cells, cells were incubated for 5 h at 20°C for synchronisation in M phase and released at 30°C in YES containing 500 μM EdU for 30 min before harvesting.

Mouse ESCs were grown on gelatin-coated dishes (0.2%) in serum + LIF conditions at 37°C with 5% CO_2_. Media was prepared by supplying DMEM-GlutaMAX-pyruvate with fetal bovine serum (15%), LIF (made in house), 1x non-essential amino acids (Gibco), 1× penicillin/streptomycin (Gibco) and 2-beta-ME (0.1 μM). Cells were passaged using Trypsin-EDTA (Gibco) or TrypLE (Gibco). Cells were routinely tested for mycoplasma contamination. To incorporate EdU, cells were pulsed in medium containing 10 μM EdU for 10 min and harvested immediately. For sample collection, media was aspirated, plates washed 2x with ice-cold PBS. Cells were scraped in a cold room and collected by centrifugation, followed by nuclei isolation. Nuclei were aliquoted, snap-frozen and stored at −80°C.

### Method details

#### Plasmid constructions

The Phusion DNA polymerase (Thermo Fischer Scientific) and oligonucleotides purchased from IDT were used for PCR amplification prior to cloning. Cloning of PCR amplicons into appropriate vectors was carried out by Gibson Assembly (New England Biolabs) or using the T4 DNA ligase (Roche) as indicated. Mutations untraceable by PCR were sequenced by Sanger sequencing (Eurofins Genomics). Used oligonucleotides are listed in Table S2.

The pSC20 plasmid containing *mrc1*^*+*^ and *ura4*^*+*^ was constructed by amplifying genomic DNA of strain 968 using oligonucleotides GTO-1652 and GTO-1653 and cloning the product into pUC8 containing the 1.8-kb *Hind*III *ura4*^*+*^ fragment (pGT189). pGT189 was amplified using oligonucleotides GTO-1650 and GTO-1651 and combined with the *mrc1* amplicon by Gibson assembly (NEB). Oligonucleotides GTO-1654 and GTO-1655 were used to amplify pSC20 excluding the NTHBS domain (deleted aa 396–535) to create pSC21. Oligonucleotides GTO-1781 and GTO-1782 were used to introduce the S353A mutations followed by oligonucleotides GTO-1783 and GTO-1784 to introduce the S797A mutation to create plasmid pAW2. Oligonucleotides GTO-2035 and GTO-2036 were used to introduce the M755A, F758A, and L774 mutations to create plasmid pSC67. Oligonucleotides GTO-2018 and GTO-2019 were used to introduce the R784A, K785A, R786A, N787A mutations in pSC20 to create pSC43 and in pSC40 to create pSC48.

To create the pSC13 plasmid containing the *mrc1ΔHBS-GFP-3HA* fusion the *mrc1-GFP- 3HA:kanMX* sequence including ±600 bp flanking DNA was amplified from FY14587^124^ using the primers GTO-1670 and GTO-1671 and cloned into pJET1.2. To introduce mutations in *mrc1* oligonucleotides were designed that either exclude parts of the ORF or introduce nucleotide substitutions in pSC13. To make the *mrc1-DSE/A* substitutions, oligonucleotides GTO-1894 and GTO-1895 were used to create pSC37. To make the *mrc1- KAF* substitutions, GTO-1896 and GTO-1897 were used to create pSC38. To take out the HBS domain (deleted aa 782–879), oligonucleotides GTO-1672 and GTO-1673 were used to create pSC22.

Plasmids containing the GBP gene fused with *S. pombe* genes were constructed by inserting the promoter and coding regions of the genes in question into rDNA2 (pDUAL based plasmid^125^ containing GBP fused with RFP under the control of the *nmt1* promoter) lacking RFP. RFP was removed from rDNA2 using the oligonucleotides GTO-1676 and GTO-1677 to construct plasmid pSC18. The pSC18 plasmid was opened using GTO-1674 and GTO-1675 excluding the *nmt1* promoter and combined with the *pob3* coding sequence and upstream region amplified by oligonucleotides GTO-1682 and GTO-1683 from genomic DNA of strain 969 by Gibson assembly to construct pSC26. Oligonucleotides GTO-1704 and GTO-1705 as well as GTO-1706 and GTO-1707 were used to amplify pSC26 excluding aa 2–31 and 332–361 followed by self-ligation using the T4 ligase to create plasmids pSC32 and pSC33, respectively.

The pPA52 plasmid containing the *mcm2* HBD (*mcm2-HBD*) was constructed by amplifying the *mcm2-HBD* using oligonucleotides mcm2-HBD F and mcm2-HBD R and ligating SpeI-XbaI-digested amplicon with SpeI-XbaI-digested pGT189. Mutations Y80A and Y89A (*mcm2-2A*) were introduced using oligonucleotides mcm2-HBD_mut F and mcm2-HBD_mut R to create pPA53.

#### *S. pombe* strain constructions

*S. pombe* strains containing seamless *mrc1ΔNTHBS*, *mrc1-S353A-S797A, mrc1-K833A-F835A* (KAF), *mrc1-M755A-F758A-L774A* (MFL), *mrc1-R784A-K785A-R786A-N787A* (RKRN), and *mrc1-R784A-K785A-R786A-N787A-K833A-F835A* (RKRN-KAF) alleles were constructed by transforming the PAM73, PG3950, HU52, and SC344 strains with the pSC21, pAW2, pSC40, pSC67, pSC43, and pSC48 plasmids digested with *Age*I (pSC21) and SpeI (rest), respectively. *S. pombe* strains containing seamless *mcm2-2A* mutations were constructed by transforming PG3950 with the pPA53 plasmid digested with *BstE*II. Ends-in recombination of the transforming DNA containing the *ura4*^*+*^ gene confers uracil prototrophy. Ura^+^ colonies were isolated followed by a subsequent isolation of FOA^R^ to select for excision of the *ura4*^*+*^ gene. FOA^R^ isolates were tested by PCR to confirm the *mrc1ΔNTHBS* allele using oligonucleotides GTO-1660 and GTO-1661, to confirm the *mrc1-2ST/A* allele using oligonucleotides GTO-760 and GTO-761, and to confirm the *mrc1-KAF*, *mrc1-MFL*, *mrc1-RKRN*, and *mrc1-RKRN-KAF* alleles using oligonucleotides GTO-1484 and GTO-1485, followed by Sanger sequencing.

*S. pombe* strains containing the *mrc1ΔHBS-GFP-3HA*, *mrc1-DSE-GFP-3HA*, and *mrc1- KAF-GFP-3HA* alleles were constructed by transforming the SC175 strain with the pSC22 plasmid or the HU52 strain with the pSC37 and pSC38 plasmids all digested with *Not*I. When integrated, the transforming DNA will add the KanMX cassette downstream of the *mrc1* locus conferring G418 resistance. G418^R^ transformants were isolated and tested by PCR for the integration of the KanMX cassette downstream of *mrc1* using oligonucleotides GTO-1686 and GTO-1687. The deletion of HBS was verified by PCR using oligonucleotides GTO-1484 and GTO-1485, while the DSE/A and KAF mutations were verified by sequencing a PCR fragment amplified by GTO-760 and GTO-761.

*S. pombe* strains containing *pob3-GBP*, *pob3Δ(2–31)-GBP*, and *pob3Δ(332–361)-GBP* promoter, coding, and terminator sequences at the *leu1* locus were constructed by transforming the SC175 strain with *Not*I-digested pSC26, pSC32, and pSC33, respectively. Integration of the transforming DNA at the *leu1* locus confers leucine prototrophy. Leu^+^ colonies were isolated.

#### Mouse ESC genome editing

CLASPIN mutated cells were generated by CRISPR-Cas9 using the SpCas9(BB)-2A-Puro (PX459) V2.0 plasmid (Addgene #62988) as described in^126^ with CLASPIN_sgRNA#1 (Table S2), which target the CLASPIN gene at the beginning of exon 19. Cells were transfected using Lipofectamine 3000 reagent (Invitrogen) using 0.5 μg of sgRNA-plasmid. Cells were sparsely seeded on a 10 cm dish 24 h posttransfection and selected with Puromycin (2 μg/mL) for 48 h. Thereafter, cells were expanded and genotyped with CLASPIN primer F and CLASPIN primer R (Table S2). Positive clones were analyzed by Sanger sequencing (Integrated DNA Technologies).

#### Acquisition and analysis of fluorescence images

To measure the fraction of (Kint2)YFP and (EcoRV)mCherry expressing cells 3 to 6 liquid cultures were set up in parallel for each strain. The cultures were inoculated in 1 or 2 mL EMM2 minimal medium supplemented as needed and incubated with vigorous agitation at 33°C unless otherwise noted. To measure the fraction of cells undergoing haploid meiosis a patch of cells was prepared on MSA medium and incubated as indicated in figure legends. Cells were scraped and suspended in water, then pelleted and fixed with 70% ice-cold ethanol and washed twice in PBS. Cells were re-suspended in PBS and stained with Hoechst. Fluorescence images were acquired with an Xcyto 10 Quantitative Cell Imager using the Xcytoview software by placing 15 or 50 μL culture onto a disposable 2-chamber or 6-chamber glass slide and imaging 72 or 17 fields. Exposure times of 1 s was used for both YFP and mCherry. Images were analyzed with the Xcytoview (version 1.1.11.0) software and visually inspected to introduce corrections when necessary. Files retrieved in.fcs format were further analyzed and plotted in R. A Nikon Ti Eclipse microscope was used for time-lapse microscopy and other fluorescence images were acquired with a Zeiss Axioplan microscope, in both cases using 100× objectives. Flow cytometry.fcs files extracted from Xcytoview were imported and visualised in R using packages flowCore (version 2.10.0), ggcyto (version 1.26.4), and ggpubr (version 0.6.0).

#### Immunofluorescence of mES cells

mES cells were pulsed in EdU-containing media (10 μM) for 10 min, washed with cold PBS and immediately fixed for 15 min in 4% PFA at room temperature and stored in PBST (PBS with 0.3% Triton X-100). Click-it was performed using Click-iT Plus Alexa Fluor 647 Picolyl Azide Toolkit (Thermo Scientific) to manufacturer’s protocol. After three washes, samples were stained with DAPI (1:10 000) in PBST. Images were acquired with a ScanR high-content screening microscope (Olympus). Automated and unbiased image analysis was carried out with the ScanR analysis software (version 2.8.1). Individual cells were identified based on DAPI staining and mean and total pixel intensity was measured for each channel. Data were exported and processed using Spotfire software (version 12.1.1; Tibco). Visualization of results was done using using R (v4.2.2) in RStudio (v2023.12.1.402). Cell cycle gates were defined using mean EdU and total DAPI intensities.

#### BrdU immunoprecipitation and next generation sequencing

Cells were grown, synchronized, and labeled with BrdU as indicated above. For BrdU immunoprecipitation (BrdU-IP), 100 mL cells (at 10^7^ cells/ml) were washed with 20 mL ice-cold 100 mM EDTA 0.1% NaN_3_ and cell pellets were moved to −80°C. Cell pellets were thawed, resuspended in 5 mL 0.5% westase solution and incubated in the dark for 4 h at 30°C. Cells were washed once in 5 mL Y1 buffer (Qiagen) and resuspended in 5 mL G2 buffer (Qiagen) and treated with 0.2 mg/mL RNase A and 0.4 mg/mL Proteinase K overnight at 55°C. Total DNA was purified using the Blood and Cell culture DNA Midi kit (Qiagen) and eluted in 1x TE. DNA was sheared to 300–600 bp using a Branson Digital Sonifier (4x, 15 s 15% amplitude, on ice 1 min in between). DNA (200 μL) was denatured for 10 min at 100°C and quickly mixed 100 μL ice-cold 2x PBS pH 7.4. Denatured DNA was added to ice-cold anti-BrdU bound magnetic beads (prepared by incubating 6 μg anti-BrdU (MBL, 2B1) with 40 μL anti-mouse IgG1 Dynabeads (Thermo Fischer Scientific) at 4°C overnight) and incubated in the dark on a rotor for 4 h at 4°C. Subsequently, the Dynabeads were separated using a magnetic rack and washed twice in 1 mL ice-cold lysis buffer (50 mM HEPES-KOH pH 7.5, 140 mM NaCl, 1 mM EDTA, 1% Triton X-100, 0.1% sodium deoxycholate), twice in 1 mL ice-cold high salt lysis buffer (50 mM HEPES-KOH pH 7.5, 500 mM NaCl, 1 mM EDTA, 1% Triton X-100, 0.1% sodium deoxycholate), twice in 1 mL ice-cold wash buffer (10 mM Tris–HCl pH 8.0, 250 mM LiCl, 0.5% NP-40, 0.5% sodium deoxycholate, 1 mM EDTA), and once in 1 mL ice-cold 1xTE. Immunoprecipitated DNA was eluated in 40 μL 1% TES (1% SDS, 1x TE) for 15 min at 65°C. Eluates were treated with 0.5 mg/mL Proteinase K for 1 h at 37°C and 1x TE was added to eluates to a total volume of 100 μL. DNA was purified using the QIAquick PCR purification kit (Qiagen). Input and immunopurified DNA was end-repaired, ligated to sequencing adaptors and amplified according to the protocol of the NEBNext Ultra II DNA Library Prep Kit for Illumina and NEBNext Multiplex Oligos for Illumina (NEB). The amplified libraries were sequenced on an Illumina NextSeq1000 instrument to generate paired-end reads of 50 bp.

#### Chromatin immunoprecipitation and xSCAR sequencing in *S. pombe*

Cells were grown, synchronized, and labeled with EdU as indicated above. Chromatin immunoprecipitation (ChIP) for ChIP-seq and xSCAR-seq was performed according to^127^ with minor modifications. Briefly, 50 mL cells (OD_600_ = 1.2) were crosslinked with 1% formaldehyde (final concentrations) for 15 min at room temperature with constant rotation and quenched with 130 mM Glycine for 5 min at room temperature, washed 2x with 15 mL ice-cold PBS and snapfrozen in liquid Nitrogen (N_2_). Cell pellets were thawed on ice and resuspended in 400 μL ice-cold lysis buffer (50 mM HEPES-KOH pH 7.5, 140 mM NaCl, 1 mM EDTA, 1% Triton X-100, 1 mM Phenylmethanesulfonyl fluoride (PMSF), and 1× cOmplete Protease Inhibitor Cocktail (Roche)). Roughly 150 μL Silica beads were added to each cell suspension and cells were lysed at 4°C using a Mini-Beadbeater-24 for 1 min at 6.5 m/s with 2 min incubation on ice between each round. Thereafter, the crude lysate was collected by puncturing the tubes and centrifugation (300 g, 1min, 4°C). Volumes of the lysates were adjusted to 1.5 mL with complete lysis buffer and sonicated in 15 mL Bioruptor Pico tubes (Diagenode) for three rounds at 4°C (10 cycles, 30 s on/30 s off, “High” mode) using the Bioruptor Pico system (Diagenode). Samples were cooled on ice for 5 min between each round. Afterward, lysates were cleared twice by centrifugation (15′700 g, 4°C) for 5 and 15 min, respectively. After saving 50 μL of lysate for the input control, the cleared lysate was used for immunoprecipitation using 5 μg anti-H3K9me2 (Abcam, ab1220), 5 μg anti-H3K36me3 (Abcam, ab9050) or 5 μg anti-H4K20me0 (Abcam, ab227804) and incubated on a rotor overnight at 4°C.

The next day, 30 μL Dynabeads (protein A and protein G Dynabeads, Invitrogen) were added for 2 h at 4°C. Subsequently, the Dynabeads were separated using a magnetic rack and washed three times with 1 mL ice-cold lysis buffer, once with 1 mL ice-cold wash buffer (10 mM Tris-HCl pH 8.0, 250 mM LiCl, 0.5% Nonidet P40 (NP-40), 0.5% Na-deoxycholate, 1 mM EDTA) and once with 1 mL ice-cold 1× TE. The ChIPs were eluted first in 100 μL 1% TES for 10 min at 65°C. and a second time in 150 μL 0.67% TES (0.67% SDS, 1× TE; 5 min, 65°C) and eluates combined. Inputs were adjusted to 250 μL by adding 200 μL elution buffer. All samples were decrosslinked overnight at 65°C, then treated with 40 μL RNase A (1 h, 37°C) and 60 μL Proteinase K (1 h, 37°C) and DNA precipitated using 150 mM NaCl and 1 volume isopropanol (15 min, 25°C), cleaned up using 30 μL AMPure XP beads, and eluted in 50 μL elution buffer (10 mM Tris-HCl pH 8.0, 1 mM EDTA).

For ChIP-seq, 40 μL of the ChIP eluates and 10 μL of their corresponding inputs were converted into libraries using KAPA Hyper prep protocol (Roche) and PCR-amplified for 5–8 cycles. The resulting PCR product was purified using a two-sided AMPure XP beads step (0.85x – 0.56x) and sequenced paired-end on an Illumina NextSeq2000.

For xSCAR-seq, sample preparation was performed according to^58^ with minor modifications. Briefly, 45 μL of ChIP eluates and 15 μL of their corresponding inputs were converted into libraries using the KAPA Hyper prep protocol and purified using AMPure XP beads with a 0.9x ratio. Up to 4 samples (from the 4 different strains assessed) were pooled to simultaneously undergo the Click-IT reaction (final concentration: 1x Click-IT buffer, 0.5 mM Biotin-TEG-Azide, 0.5 mM THPTA, 0.1 mM CuSO4, 10 mM Sodium Ascorbate) for 30 min, 25°C. Pooled, clicked samples were purified using AMPure XP beads (0.9x ratio), eluted and subjected to immunoprecipitation with Dynabeads MyOne Streptavidin (Invitrogen, 65602) with a 30 min incubation step on a rotator at 25°C. Bound biotinylated DNA was subjected to stringent washes: 4x washes with 1x BW (5 mM Tris-HCl pH7.5, 0.5 mM EDTA, 1 M NaCl, 0.05% Tween 20), 1x wash with 2x BW, 3x washes with Alkaline wash buffer (100 mM NaOH, 0.05% Tween 20), 2x washes with 1x BW, 1x wash with 10 mM Tris-HCl pH 8.0 and eluted in 20 μL elution buffer. Bound, stranded, biotinylated DNA was PCR-amplified for 12 cycles, purified using a two-sided AMPure XP beads step (0.85x – 0.56x) and sequenced paired-end on a NextSeq2000.

#### SCAR-seq in mES cells

A step-by-step protocol is available.^83^ Briefly, nascent SCAR-seq samples were prepared from WT and CLASPIN mutant cells in two biological replicates for each histone PTM. Cells were pulsed in EdU-containing media (10 μM) for 10 min and harvested immediately. For sample collection, media was aspirated, plates washed 2× with ice-cold PBS. Cells were scraped in a cold room and collected by centrifugation, followed by nuclei isolation. Nuclei were aliquoted, snap-frozen and stored at −80°C until further use. For MNase digest, nuclei were counted manually using Kova Glasstic Slides and 2 U MNase (Worthington) were added per 1 × 10^6^ nuclei. Digests were performed at 30°C for 20 min. For native ChIP, 45 μg of chromatin was used per sample and incubated with antibodies in a total volume of 600 μL overnight at 4°C with H3K27me3 antibody (Cell Signaling, 9733) or H4K20me0 antibody (Abcam, ab227804). Magnetic beads (anti-rabbit IgG Dynabeads, invitrogen) were added the next morning and samples were incubated for 2 h. After three washes each with ice-cold RIPA buffer and RIPA 0.5 M NaCl buffer, DNA was eluted and purified using the MinElute Reaction Cleanup kit (Qiagen). Mononucleosomal-sized fragments were isolated by double sided size selection with AMPure XP beads (Beckman Coulter). EdU-labelled DNA fragments were biotinylated using Click-iT chemistry using Biotin-TEG-Azide (Berry & Associates). Libraries were prepared using the KAPA Hyper Prep Kit (Roche). Biotinylated fragments were captured using Dynabeads MyOne Streptavidin (Invitrogen) and EdU strands were purified by performing NaOH washes. Libraries were amplified in 11 PCR cycles. Libraries with mononucleosomal-sized inserts were isolated by double-sided size selection with AMPure XP beads (Beckman Coulter), followed by a second clean-up with 1.0x AMPure XP beads. Fragment distribution of libraries was checked on a TapeStation Instrument (Agilent). Stranded input samples were prepared in parallel with SCAR-seq samples. Samples were sequenced paired-end (57 bp and 56 bp) with Unique molecular identifiers (UMIs) on a NextSeq2000 instrument (Illumina).

#### Protein expression and purification

Full-length 6xHis-Mrc1-3xFLAG was expressed by cloning the Mrc1 ORF amplified with Mrc1(Bam)-fwd and -rev primers at the BamHI site of ver3.4 vector.^128^ The plasmid was transfected into 293FT cells and 6xHis-Mrc1-3xFLAG was purified using ANTI-FLAG M2 Affinity Gel (SIGMA A2220) as described.^22^^,^^128^ The protein was eluted by DYKDDDDK (x3) Tag Eluting Peptide (ABGENT BP1013i) and dialyzed against the buffer (25 mM Hepes-KOH [pH7.6], 10% glycerol, 100 mM potassium glutamate, 1mM EDTA, and 1mM DTT) using Spectra/Por 2 Dialysis Membrane (MWCO: 12–14 kDa, Spectrum Laboratories, Inc.) overnight at 4°C to remove the eluting peptide.

Mrc1(674–879) WT and mutant and SUMO-HA-Mcm2(34–165) fragments were produced as gBlocks (IDT), cloned into a modified pNIC-Bsa vector (SGC) with an N-terminal 6xHis tag and expressed in *E. coli*. The proteins were initially purified on a HisTrap affinity column. SUMO was cleaved using the SUMO protease. Proteins were further purified on a HiLoad 16/600 Superdex 200 Column (Mrc1) and HiLoad 16/60 Superdex 75 column (Mcm2) (Cytiva).

Histone H3.1/H4 used in Figure 5D were prepared by denaturing lyophilized histones and refolding as described in.^129^ Constitutively cross-linked H3-H4 tetramers were prepared by incubating 25 μM Xenopus laevis H3-C110A-K115C - H4 WT mutant histones in 20 mM HEPES pH 7.5, 1 M NaCl, 1 mM EDTA in 50 μM BMOE (bismaleimidoethane) for 1 h at room temperature. Cross linking was quenched by addition of 50 mM DTT to a final concentration of 10 mM DTT and 20 μM XL-(H3-H4)_2_. Cross linking was assayed by SDS-PAGE without boiling of samples. *X. laevis* H3-C110E-L126A-I130A - H4 were used as constitutive dimers.^95^

#### HA pull-downs

Ten μL of Anti-HA-tag mAB-Magnetic Beads (M180-11, MBL) per sample were washed with 5 mg/mL BSA (A3059; Sigma-Aldrich) in PBS containing 0.1% Tween 20 for 30 min to prevent non-specific binding. The beads were washed with CSK-500 mM NaCl buffer (50 mM Hepes-NaOH [pH7.5], 500 mM NaCl, 1 mM EDTA, 1 mM EDTA, 2 mM MgCl2, 10% (v/v) glycerol, 1 mM DTT, 0.1 mM ATP, 0.5 mM 1 mM sodium orthovanadate (V), 50 mM sodium fluoride, 1×cOmplete, EDTA-free (05056489001; Roche), 0.1% Triton X-100). The beads were first mixed with 40 pmol HA-Mcm2 (25 kDa) in 100 μL CSK-500 mM NaCl buffer and placed on a rotating wheel for 1 h at 4°C. Then, 1.5 pmol of Mrc1-FLAG (114 kDa) and 19 pmol of human histone H3.1-H4 tetramer (Platform Project for Supporting Drug Discovery and Life Science Research, AMED) were added to the bead suspensions in this order. After incubation for 3 h at 4°C with rotation, the beads were washed with CSK buffer 4 times and heated at 95°C for 3 min in 50 μL Laemmli SDS sample buffer. 10 μL of the sample was run on 4–20% SDS-PAGE gel and blotted onto Immoblion PVDF membrane (IPVH00010; Millipore), which was blocked with Blocking one (03953-95; NACALAI TESQUE, INC.) before incubation with an antibody. FLAG and HA were detected, respectively, with monoclonal anti-FLAG M2 antibody horseradish peroxidase (HRP) antibody (A8592; Sigma-Aldrich) and Direct-Blot HRP anti-HA.11 Epitope Tag antibody (901520; BioLegend). Histone H3-H4 was detected by staining with Coomassie Brilliant Blue (CBB).

#### His pull-downs

6xHis-Mrc1(674–879) was incubated at 1 μM with 1 μg anti-6X His-Tag (Abcam, ab18184) in 135 μL binding buffer (20 mM Tris, pH 7.5, 0.4 M NaCl, 5% Glycerol) for 1 h at 4°C. 15 μL of Dynabeads Protein G (Invitrogen) was added and the suspension was incubated for 1 h at 4°C on a rotating wheel. Beads were washed once with 500 μL binding buffer followed by addition of 150 μL binding buffer containing 0.5 μM X. laevis H3-H4 dimers or cross-linked H3-H4 tetramers and incubation for 1 h at 4°C on a rotating wheel. Beads were washed four times with 500 μL wash buffer (20 mM Tris, pH 7.5, 0.6 M NaCl, 1% Triton X-100) followed by elution in 20 μL 1xLSB, heated at 95°C for 5 min. Samples were analyzed by SDS-PAGE and immunoblotting onto a nitrocellulose membrane (Cytiva) using anti-His (Abcam, ab18184) and anti-H4K20me0 (Abcam, ab227804). CW800 anti-rabbit or CW680 anti-mouse secondary antibodies were used for detection (LI-COR).

#### Crude cell extracts from *S. pombe*

Cell extracts were prepared by washing cells pellets once in 500 μL H2O, and re-suspending in 100 μL H2O. The cell suspension was boiled for 5 min and quickly transferred to ice. 100 μL 2x SDS sample buffer was added. Roughly 150 μL silica beads were added and the cells were lysed at 4°C using a Mini-Beadbeater-24 for 3 × 1 min at 6.5 m/s with 2 min incubation on ice between each round. Thereafter, the crude lysate was collected by puncturing the tubes and centrifugation (300 g, 1 min, 4°C) and lysate was boiled for 5 min and analyzed by SDS-PAGE and immunoblotting onto a nitrocellulose membrane (Cytiva) using anti-FLAG (Sigma-Aldrich, M2) and anti-TAT1. CW680 anti-mouse secondary antibody was used for detection (LI-COR).

#### Chromatin fractionation of *S. pombe* cell extracts

Cells pellets were washed once in sorbitol buffer (1.2 M sorbitol, 1 mM DTT, 10 mM HEPES-KOH, pH 7.6, 40 mM potassium glutamate, 1 mM MgCl_2_, 1 mM EGTA, 1 mM EDTA, 0.5x cOmplete Protease Inhibitor Cocktail (Roche), and 1 mM PMSF) and treated with lyticase for 30 min. Spheroplasts were washed three times with sorbitol buffer, resuspended in 2x volume extraction buffer (10 mM HEPES-KOH, pH 7.6, 40 mM potassium glutamate, 1 mM MgCl_2_, 1 mM EGTA, 1 mM EDTA, 1 mM DTT, 1x cOmplete Protease Inhibitor Cocktail (Roche), 1 mM PMSF) containing 1% Triton X-100, and kept on ice for 20 min. Unlysed spheroplasts were pelleted by centrifugation at 550*g* for 1 min at 4°C and supernatant was centrifuged at 20000 g for 10 min at 4°C to yield Triton X-100 soluble supernatant and chromatin-enriched pellet. The pellets were washed once with 500 μL extraction buffer. Fractions were analyzed by SDS-PAGE and immunoblotting onto a nitrocellulose membrane (Cytiva) using anti-FLAG (Sigma-Aldrich, M2) and anti-TAT1. CW680 anti-mouse secondary antibody was used for detection (LI-COR).

#### Cell extracts for mESCs

Cell extracts were prepared by washing cell pellets twice in ice-cold PBS and hypotonic lysis buffer (10 mM Tris pH 7.4, 2.5 mM MgCl_2_, 0.5% Nonidet P40, 5 mM NaF and 10 mM β-Glycerolphosphate, 0.1 mM PMSF, 10 μg/mL Leupeptin, 10 μg/mL, Pepstatin A, 100 ng/mL Trichostatin A, and 0.2 mM Na_3_VO_4_). Cells were spun down at 600 g for 3 min at 4°C and supernatant was removed. Pellet was resuspended in extraction buffer (300 mM NaCl, 0.5% NP-40, 50 mM Tris.HCl pH 7.4, 0.2 mM EDTA, 1 mM MgCl_2_, 5% glycerol, 5 mM NaF, 10 mM β-Glycerolphosphate, 0.1 mM PMSF, 10 μg/mL Leupeptin, 10 μg/mL Pepstatin A, 100 ng/mL Trichostatin A, 0.2 mM Na_3_VO_4_. Extract was treated with 1000 U Benzonase (E1014, Millipore) for 1 h at 4°C. Supernatants clarified by centrifugation at 16,000 g for 5 min). Western blotting was performed as described.^55^ Western blots were performed with the following antibodies: CLASPIN (Bethyl Laboratories, # A300-266A) and MCM2 (Cell Signaling, #3619). CW800 anti-rabbit secondary antibody was used for detection (LI-COR).

#### Alphafold analysis

Structures of *S. pombe* complexes comprising full-length Mrc1 (UniProt ID: Q9P7T4) and Mcm2 (P40377) bound to an H3/H4 (P09988, P09322) histone tetramer, full-length Mrc1 bound to an H3/H4 histone dimer and H3/H4 tetramer were predicted with Alphafold multimer v.2.3^90^^,^^91^ using up-to-date databases [2023-01-06] with default parameters.^130^ From the 25 models generated each run, only the top ranking model was considered. Full-length amino acid sequences were retrieved from UniProt.^131^ Mrc1-Mcm2 and Mcm2-H3/H4 histone interaction interfaces in the best scoring Mrc1-Mcm2-H3/H4 tetramer model were compared to existing experimental structures (PDB: 8B9A^4^ and 5BNV^51^) using Swiss Model^132^ structure validation web tool (https://swissmodel.expasy.org/assess, DockQ^133^ and QS-scores^134^). As the AF models were generated for full-length protein sequences, truncated models were used to generate the DockQ/QS scores to minimize the influence of experimentally unresolved residues (see Figure S7). Mrc1 short helix fragment 323–339 in PDB: 8B9A has contacts solely with TOF1 protein not present in our AF predictions and therefore was not considered for the validations. Structures were visualized in PyMOL v1.2r3pre^110^ and ChimeraX 1.7^127^. All the AF models included in the manuscript, as well as the validation results, can be retrieved from "https://sid.erda.dk/sharelink/hkAiSCzwqV".

### Quantification and statistical analysis

#### BrdU-IP

BrdU-IP and Input fastq sequences were mapped to the *S. pombe h*^90^ contig using Bowtie2 (v2.5.0) applying the --no-mixed and --no-discordant alignment options.^112^ Duplicated reads were removed using samtools.^113^ Peaks were called using model-based analysis of ChIP-seq^114^ (MACS2 version 2.2.7.1). Coverage plots were visualized with the Integrated Genome Visualization software.^115^

#### ChIP-seq and xSCAR-seq

Fastq files were processed using the Galaxy Pipeline usegalaxy.eu.^116^ In brief, fastq files were aligned to the *S. pombe h*^90^ contig using bowtie2 (v2.5.0) with standard parameters applied.^112^ Duplicated reads were removed using RmDup from samtools.^113^ Resulting bam files were converted into bigwig files, were input normalized using deeptools^117^ bamCompare command (with parameters: --normalizeUsing CPM --centerReads --maxFragmentLength 1000 --scaleFactorsMethod None --pseudocount 1) and visualized using the IGV browser.^115^

For mESC SCAR-seq, reads were processed, mapped, and deduplicated using UMIs to bam files as described previously.^49^ For both fission yeast and mESCs, the script from^49^ was used to generate bigwig files with their local histone partitioning signal. Briefly, the resulting processed bam files were split into forward and reverse strands according to the SAM flag, using samtools view (version 1.5) -F 20 and -f 16, respectively. Then, for each strand the xSCAR normalized signal (CPM) was computed in 500b bins (1kB bins for mESCs SCAR-seq) and smoothed in a uniform blur considering the neighboring 5 bins (30 bins for mESCs SCAR-seq) on each side. For each window, the signal from its corresponding xSCAR input was subtracted and negative values were set to zero. The final partition score for each window was calculated as:

Partition = (F - R)/(F + R) where F and R correspond to the number of normalized and input-corrected reads for the forward and reverse strand, respectively. The partition value relates to the ratio of histones with a specific modification being segregated to the nascent forward (Partition >0) or nascent reverse (Partition <0) strand within each window respectively. In the case xGen UDI-UMI adapters are used for xSCAR library preparation, please note that the complementary strand will be sequenced ("https://eu.idtdna.com/pages/support/faqs/can-the-xgen-unique-dual-index-umi-adapters-be-used-for-rna-seq"). This results in a seemingly mirrored Partition ratio (R-F)/(R + F) that should be corrected by multiplication with −1.

External datasets

Replication origins (*S. pombe)*: GSE62108^89^

Replication origins (mESCs): GSE117274^49^

To calculate the xSCAR density plots, the script from^83^ was used. Briefly, the read number of xSCAR ChIP samples (H3K36me3 or H4K20me0) was divided by the read number of the corresponding clicked Inputs and stratified by either leading or lagging-strand orientation. Only reads mapping to the 2.5kB surrounding region of replication initiation centers (+-2.5 kB) were considered and normalized to read depth (CPM).

To visualize biases according to their replication fork orientation, replication origins were downloaded from.^89^ The bedgraph file with all annotated origins was remapped to fit the coordinates of the *S. pombe h*^90^ contig and was filtered for a firing score of >20 to remove low-firing origins.

Heatmaps and Lineplots were generated using Seqplots^118^ and genome browser snapshots of xSCAR partitioning were obtained from the IGV browser.^115^ H3K9me2 ChIP occupancy was visualised in R using the Gviz package (version 1.46.1).

Heatmaps for H3K9me2 occupancy were generated using deeptools MultiBigWigSummary across heterochromatic regions taken from^135^ and gplot heatmap.2.

#### Statistical analysis

Statistical analyses of mCherry expression data were carried out in R (v4.2.2). The Shapiro-Wilks test for normality was used to determine data distribution (passed at *p* > 0.05). For datasets in which at least one sample did not pass the test for normality non-parametric tests were applied. Multiple comparison testing by ANOVA was followed by the Tukey HSD post-hoc test, while Kruskal-Wallis testing was followed by pairwise Nemeyni’s rank-sum test. The specific tests applied in individual experiments and specific *p* values are indicated in figure legends. ^∗∗∗^*p* < 0.001. For xSCAR density analysis the unpaired Wilcoxon test was used.

## Supplemental Information

Document S1. Tables S1 and S2, related to STAR Methods
