# Supplementary material for: The fork protection complex promotes parental histone recycling and epigenetic memory
Source: Cell. 2024 Sep 5;187(18):5029–5047.e21. doi: 10.1016/j.cell.2024.07.017 (PMC11383432; doi:10.1016/j.cell.2024.07.017)
Supplement: Document S1. Tables S1 and S2, related to STAR Methods [file mmc1.pdf]

Cell, Volume *187*

Supplemental information

The fork protection complex promotes parental histone recycling and epigenetic memory

Sebastian Jespersen Charlton, Valentin Flury, Yutaka Kanoh, Aitana Victoria Genzor, Leonie Kollenstart, Wantong Ao, Peter Brøgger, Melanie Bianca Weisser, Marek Adamus, Nicolas Alcaraz, Charlotte M. Delvaux de Fenffe, Francesca Mattiroli, Guillermo Montoya, Hisao Masai, Anja Groth, and Geneviève Thon

Table S1: Strains used in this study, related to STAR Methods.

Strain	Genotype
AW16	h^{90} (XmnI)::ura4^{+} mat2-P kint2::YFP mat3-M(RV)::mCherry mrc1-S353A-S797A ura4-D18
HU43	h^{-} leu1-32 ura4-D18 ade6-DN/N
HU52	h^{90} mat3-M (EcoRV)::ade6^{+} leu1-32 ura4-D18 ade6-DN/N
LJ102	h^{90} mat3-M (EcoRV)::ade6^{+} (BlpI)::LEU2 mrc1Δ::kanMX4 leu1-32 ura4-D18 ade6-DN/N
KT1398	h^{-} leu1-32 ura4-D18 mrc1-S604A-T645A-T653A-5xFLAG:kan^{R}
MS563	h^{-} leu1-32 ura4-D18 mrc1Δ(782-879)
MS715	h^{-} nda3-KM311 leu1-32 ura4-D18 mrc1-T238E-S395E-3xFLAG:kan^{R}
PAM01	h^{+} mrc1Δ::kanMX4 leu1-32 ura4-D18
PAM02	h^{90} (XmnI)::ura4^{+} mat2-P kint2::YFP mat3-M(RV)::mCherry mrc1Δ::kanMX4 clr4Δ::kanR ura4-D18 arg12Δ::natR
PAM05	h^{90} (XmnI)::ura4^{+} mat2-P kint2::YFP mat3-M(RV)::mCherry swi1Δ::hph1 clr4Δ::kanR ura4-D18 arg12Δ::natR
PAM08	h^{90} (XmnI)::ura4^{+} mat2-P kint2::YFP mat3-M(RV)::mCherry swi3Δ::hph1 clr4Δ::kanR ura4-D18 arg12Δ::natR
PAM10	h^{+} mrc1Δ::kanMX4 ura4-D18
PAM11	h^{+} swi1Δ::hph1 ura4-D18
PAM12	h^{+} swi3Δ::hph1 ura4-D18
PAM33	h^{90} (XmnI)::ura4^{+} mat2-P kint2::YFP mat3-M(RV)::mCherry mcm2-Y80A-Y89A ura4-D18
PAM59	h^{90} (XmnI)::ura4^{+} mat2-P kint2::YFP mat3-M(RV)::mCherry mrc1-T238E-S395E-3xFLAG:kan^{R} ura4-D18
PAM73	h^{90} (EcoRV)::mCherry ura4-D18 ade6-216 arg12Δ::NatR
PAM94	h^{90} mat2-P kint2::YFP mat3-M(RV)::mCherry mrc1Δ::kanMX4 hsk1-89:ura4^{+} ura4-D18
PAM95	h^{90} mat2-P kint2::YFP mat3-M(RV)::mCherry hsk1-89:ura4^{+} ura4-D18
PG1247	$h^{\Delta K}$ mat3-M(RV)::ura4^{+} leu1-32 ura4-D18 ade6-210
PG1599	mat1-Msmt-0 mat2-P (XbaI)::ura4^{+} leu1-32 ura4-D18 ade6-216
PG1608	mat1-Msmt-0 REIIΔ mat2-P (XbaI)::ura4^{+} leu1-32 ura4-D18 ade6-216
PG1899	h^{90} mat3-M(RV)::ura4^{+} leu1-32 ura4-DS/E ade6-210
PG3716	h^{+} ura4-D18
PG3873	h^{90} (XmnI)::ura4^{+} mat2-P kint2::YFP mat3-M(RV)::mCherry clr4Δ::kanR ura4-D18 arg12Δ::natR
PG3950	h^{90} mat3-M (EcoRV)::ade6^{+} (BlpI)::LEU2 leu1-32 ura4-D18 ade6-DN/N
PG4154	h^{90} nda3-KM311 AUR1:aur1r-Adh1-TK-Adh1-scENT1 leu1-32
SC140	h^{90} (XmnI)::ura4^{+} mat2-P kint2::YFP mat3-M(RV)::mCherry mrc1Δ(391-535) ura4-D18 ade6-216 arg12Δ::Nat^{R}
SC160	h^{90} (XmnI)::ura4^{+} mat2-P kint2::YFP mat3-M(RV)::mCherry mrc1Δ(391-535) ura4-D18
SC185	h^{90} mat3-M(RV)::mCherry mrc1Δ(782-879)-GFP-3HA:kanMX6 leu1-32
SC191	h^{90} mat3-M(RV)::mCherry mrc1Δ(782-879)-GFP-3HA:kanMX6 leu1:pob3-GBP
SC206	h^{90} mat3-M(RV)::mCherry mrc1Δ(782-879) leu1:pob3-GBP
SC238	h^{90} mrc1Δ(782-879) nda3-KM311 AUR1:aur1r-Adh1-TK-Adh1-scENT1 leu1-32
SC239	h^{90} (XmnI)::ura4^{+} mat2-P kint2::YFP mat3-M(RV)::mCherry mrc1-S604A-T645A-T653A-5xFLAG:kan^{R} ura4-D18
SC240	h^{90} (XmnI)::ura4^{+} mat2-P kint2::YFP mat3-M(RV)::mCherry mrc1Δ::kanMX4 ura4-D18
SC241	h^{90} (XmnI)::ura4^{+} mat2-P kint2::YFP mat3-M(RV)::mCherry mrc1-SUMO^{-}:kan^{R} ura4-D18
SC242	h^{90} (XmnI)::ura4^{+} mat2-P kint2::YFP mat3-M(RV)::mCherry mrc1Δ(782-879) ura4-D18
SC243	h^{90} (XmnI)::ura4^{+} mat2-P kint2::YFP mat3-M(RV)::mCherry mrc1Δ(160-284) ura4-D18
SC279	h^{90} (XmnI)::ura4^{+} mat2-P kint2::YFP mat3-M(RV)::mCherry swi3Δ::hph1 ura4-D18
SC281	h^{90} (XmnI)::ura4^{+} mat2-P kint2::YFP mat3-M(RV)::mCherry swi1Δ::hph1 ura4-D18
SC285	h^{90} mat3-M (EcoRV)::ade6^{+} mrc1-DSE/A-GFP-3HA:kanMX6 leu1-32 ura4-D18 ade6-DN/N
SC287	h^{90} mat3-M (EcoRV)::ade6^{+} mrc1-KAF/A-GFP-3HA:kanMX6 leu1-32 ura4-D18 ade6-DN/N
SC291	h^{90} mat2-P kint2::YFP mat3-M(RV)::mCherry mrc1Δ(782-879) hsk1-89:ura4^{+} ura4-D18
SC292	h^{90} (XmnI)::ura4^{+} mat2-P kint2::YFP mat3-M(RV)::mCherry mrc1-DSE/A-GFP-3HA:kanMX6 ura4-D18
SC293	h^{90} (XmnI)::ura4^{+} mat2-P kint2::YFP mat3-M(RV)::mCherry mrc1-KAF/A-GFP-3HA:kanMX6 ura4-D18
SC294	h^{90} (XmnI)::ura4^{+} mat2-P kint2::YFP mat3-M(RV)::mCherry mrc1-DSE/A hsk1-89:ura4^{+} ura4-D18
SC295	h^{90} (XmnI)::ura4^{+} mat2-P kint2::YFP mat3-M(RV)::mCherry mrc1-KAF/A hsk1-89:ura4^{+} ura4-D18
SC298	h^{90} (XmnI)::ura4^{+} mat2-P kint2::YFP mat3-M(RV)::mCherry mrc1Δ(782-879) mcm2-Y80A-Y89A
SC302	h^{90} (XmnI)::ura4+ mat2-P kint2::YFP mat3-M(RV)::mCherry nda3-KM311 AUR1:aur1r-Adh1-TK-Adh1-scENT1 ura4-D18
SC303	h^{90} (XmnI)::ura4+ mat2-P kint2::YFP mat3-M(RV)::mCherry mrc1Δ(782-879) nda3-KM311 AUR1:aur1r-Adh1-TK-Adh1-scENT1 ura4-D18
SC304	h^{90} (XmnI)::ura4+ mat2-P kint2::YFP mat3-M(RV)::mCherry mcm2-Y80A-Y89A nda3-KM311 AUR1:aur1r-Adh1-TK-Adh1-scENT1 ura4-D18
SC305	h^{90} (XmnI)::ura4+ mat2-P kint2::YFP mat3-M(RV)::mCherry mrc1Δ(782-879) mcm2-Y80A-Y89A nda3-KM311 AUR1:aur1r-Adh1-TK-Adh1-scENT1 ura4-D18
SC322	mat1-Msmt-0 REIIΔ mat2-P (XbaI)::ura4^{+} mrc1Δ::kanMX4 leu1-32 ura4-D18 ade6-216
SC324	mat1-Msmt-0 REIIΔ mat2-P (XbaI)::ura4^{+} mrc1Δ(782-879) leu1-32 ura4-D18 ade6-216
SC328	$h^{\Delta K}$ mat3-M(RV)::ura4+ leu1-32 ura4-D18 ade6-210

SC329	*mat1-Msmt-0 mat2-P (XbaI)::*$ura4^+$ *mrc1Δ::kanMX4 leu1-32 ura4-D18 ade6-216*
SC332	*mat1-Msmt-0 mat2-P (XbaI)::*$ura4^+$ *mrc1Δ(782-879) leu1-32 ura4-D18 ade6-216*
SC341	h^+ *ura4-D18 mrc1-K833A-F835A nda3-KM311 AUR1:aur1r-Adh1-TK-Adh1-scENT1*
SC378	h^{90} *mat3-M(RV)::*$ura4^+$ *mrc1Δ(782-879) leu1-32 ura4-D18 ade6-210*
SC384	h^{90} *mat2-P kint2::YFP mat3-M(RV)::mCherry mrc1-M755A-F758A-L774A AUR1:aur1r-Adh1-TK-Adh1-scENT1 ura4-D18*
SC392	h^+ *mrc1-M755A-F758A-L774A nda3-KM311 AUR1:aur1r-Adh1-TK-Adh1-scENT1*
SC395	h^{90} *(XmnI)::ura4+ mat2-P kint2::YFP mat3-M(RV)::mCherry mrc1-R784A-K785A-R786A-N787A nda3-KM311 AUR1:aur1r-Adh1-TK-Adh1-scENT1 ura4-D18*
SC396	h^{90} *(XmnI)::ura4+ mat2-P kint2::YFP mat3-M(RV)::mCherry AUR1:aur1r-Adh1-TK-Adh1-scENT1 ura4-D18 mrc1-R784A-K785A-R786A-N787A-K833A-F835A nda3-KM311*
SC397	h^{90} *(XmnI)::ura4+ mat2-P kint2::YFP mat3-M(RV)::mCherry AUR1:aur1r-Adh1-TK-Adh1-scENT1 ura4-D18 mrc1-R784A-K785A-R786A-N787A-3FLAG:kanMX6 nda3-KM311*
SC398	h^+ *mrc1-M755A-F758A-L774A-3FLAG:kanMX6 nda3-KM311 AUR1:aur1r-Adh1-TK-Adh1-scENT1*
SC399	h^{90} *(XmnI)::*$ura4^+$ *mat2-P kint2::YFP* $mrc1^+$*-3FLAG:kanMX6 mat3-M(RV)::mCherry ura4-D18*
SC400	h^{90} *(XmnI)::*$ura4^+$ *mat2-P kint2::YFP mat3-M(RV)::mCherry mrc1Δ(782-879)-3FLAG:kanMX6 ura4-D18*
SC401	h^+ *ura4-D18 mrc1-K833A-F835A-3FLAG:kanMX6 nda3-KM311 AUR1:aur1r-Adh1-TK-Adh1-scENT1*
SOM81	h^{90} *(XmnI)::*$ura4^+$ *mat2-P kint2::YFP mat3-M(RV)::mCherry ura4-D18*
SP837	h^{90} *leu1-32 ura4-D18 ade6-216*

Table S2: Oligonucleotides used in this study, related to STAR Methods.

Oligonucleotide	Sequence
GTO-760	5'-CCAGCCATATGGCATCTTTAGACGAGAACGCTG-3'
GTO-761	5'-CGTCGCGAATTCTTAGTCAAAGTCCGAGTAATTATTCAAAGAAGC-3'
GTO-1650	5'-TCAGCGTTCTCGTCTAAAGATGCCATCTGCAGCCAAGCTTGTGATATTGAC -3'
GTO-1651	5'-CGGATCCCCGGGAATTCGTAATCATG-3'
GTO-1652	5'-CATGATTACGAATTCCCGGGGATCCGTTAGTCAAAGTCCGAGTAATTATTC-3'
GTO-1653	5'-ATGGCATCTTTAGACGAGAACGCTGA-3'
GTO-1654	5'-GCTGAGCTTGATTTCAGCAAGTTGC-3'
GTO-1655	5'- CTGGAGATTCGCATGGTTCTTCGAATATGA-3'
GTO-1660	5'-GCTTGGTTGACTGTTTTGAACATAAAGAG-3'
GTO-1661	5'-GGAAGAGGAAGCTAACTCAACTGAGCC-3'
GTO-1670	5'-GCGGCCGCTTATTCTGAGGCAAATCCTAAACGATCC-3'
GTO-1671	5'-GCGGCCGCTGTTGATACGGTCGATGATGCTCTTGTC-3'
GTO-1672	5'-CATGAAGAAGAAGAGCAAATGGAAGAGG-3'
GTO-1673	5'-ACCAGTAGTAACGTCTTTCATTAGCTG-3'
GTO-1674	5'-ATGGCCGATGTGCAGCTGGTGGAGTC -3'
GTO-1675	5'-CTCTAGAGTCGATCGACCTGCAGGCATGC-3'
GTO-1676	5'-AGATCTCGACTCTAGAGGATCCGGCGC-3'
GTO-1677	5'-TCATGAGGAGACGGTGACCTGGGTCCCCTGGC-3'
GTO-1682	5'-<br>GCATGCCTGCAGGTCGATCGACTCTAGAGAAATAAAATTTGGTATACAAAATTCACG-3'
GTO-1683	5'-GACTCCACCAGCTGCACATCGGCCATCTCCGAACCCTCAGCACCAGAAGC-3'
GTO-1704	5'-CATTTTTTATAACTGGTGAAGTATTAAG-3'
GTO-1705	5'-AAGTCACCTAGCTTAGCAGAAC-3'
GTO-1706	5'-TTGACTTACAACTTCAAAAGCAGG-3'
GTO-1707	5'-GCGAATGAAGGCCAATTATATTGC-3'
GTO-1781	5'-GAATGAGGATGACATTGATGCTGAGCTGGAAATTG-5'
GTO-1782	5'-CAATTTCCAGCTCAGCATCAATGTCATCCTCATTC-3'
GTO-1783	5'-GCAGCTTTGGACGATGCTGACGATGAAGATTACAG-3'
GTO-1784	5'-CTGTAATCTTCATCGTCAGCATCGTCCAAAGCTGC-3'
GTO-1686	5'-ATAGATGAAATGTGTATACATG-3'
GTO-1687	5'-TGTGGACAGGTAATGGTTGTC-3'
GTO-1894	5'-AGCTGCAGCCAAAGCTGCAAATCCATTCC-3'
GTO-1895	5'-GCTGCTGCAGCTTACAGTAATTTGCGGCGC-3'
GTO-1896	5'-GGCCCTTTTATCACCTTCTAAGAC-3'
GTO-1897	5'-GCAGCTTTAGCTACTGTTGAAGACAG-3'
GTO-2018	5′- CGCTGCTAGAGCACCAGTAGTAACG-3′
GTO-2019	5′-GCGGCTAGGAATGGATTTGCAGC-3′
GTO-2035	5′-CCATTTCTTGATCTTTAGCGGCTTGTGCCGCTGAAGCTACCTCGCC-3′
GTO-2036	5'-ATCGTGATGAAAAGTTAGTAAAACAGGCAATGAAAGACGTTACTACTGG-3'
mcm2-HBD F	5'-GGACATCATTCATTGTTCTAGATATG-3'
mcm2-HBD R	5'-GTGCGACTAGTTACGACACCAGATA-3'
mcm2-HBD_mut F	5'-GAGCTAGATCGTGCTGACATTGAAG-3'
mcm2-HBD_mut R	5'-CAAATTTTGTTGAGCATCACTATACAG-3'
Claspin_sgRNA#1	5'-GAGGTGTCAGGCAGTGACGT-3'
Claspin primer F	5'-TTCTCCCCAATCCTGACAGCTG-3'
Claspin primer R	5'-TAGGCAAGCATCTCTCTCCAGC-3'
Mrc1(Bam) fwd primer	5'-CATCACCACCATGGATCCATGGCATCTTTAGACGAGAACGCT-3'
Mrc1(Bam) rev primer	5'-ctgcagctcgagggatccGTCAAAGTCCGAGTAATTATTCAA-3'
